# Supplementary material for: ProtFI, an efficient frailty-trained proteomics-based biomarker of aging, robustly predicts age-related decline
Source: Cell Rep Methods. 2026 Apr 10;6(5):101405. doi: 10.1016/j.crmeth.2026.101405 (PMC13198109; doi:10.1016/j.crmeth.2026.101405)
Supplement: Document S1. Figures S1–S4 and Tables S1–S3, S5, and S8–S10 [file mmc1.pdf]

Cell Reports Methods, Volume *6*

Supplemental information

ProtFI, an efficient frailty-trained proteomics-based biomarker of aging, robustly predicts age-related decline

Swier Garst, Lieke Kuiper, Erik van den Akker, Niels van den Berg, Mohsen Ghanbari, Simon Mooijaart, Marian Beekman, Marcel Reinders, P. Eline Slagboom, and Joyce van Meurs

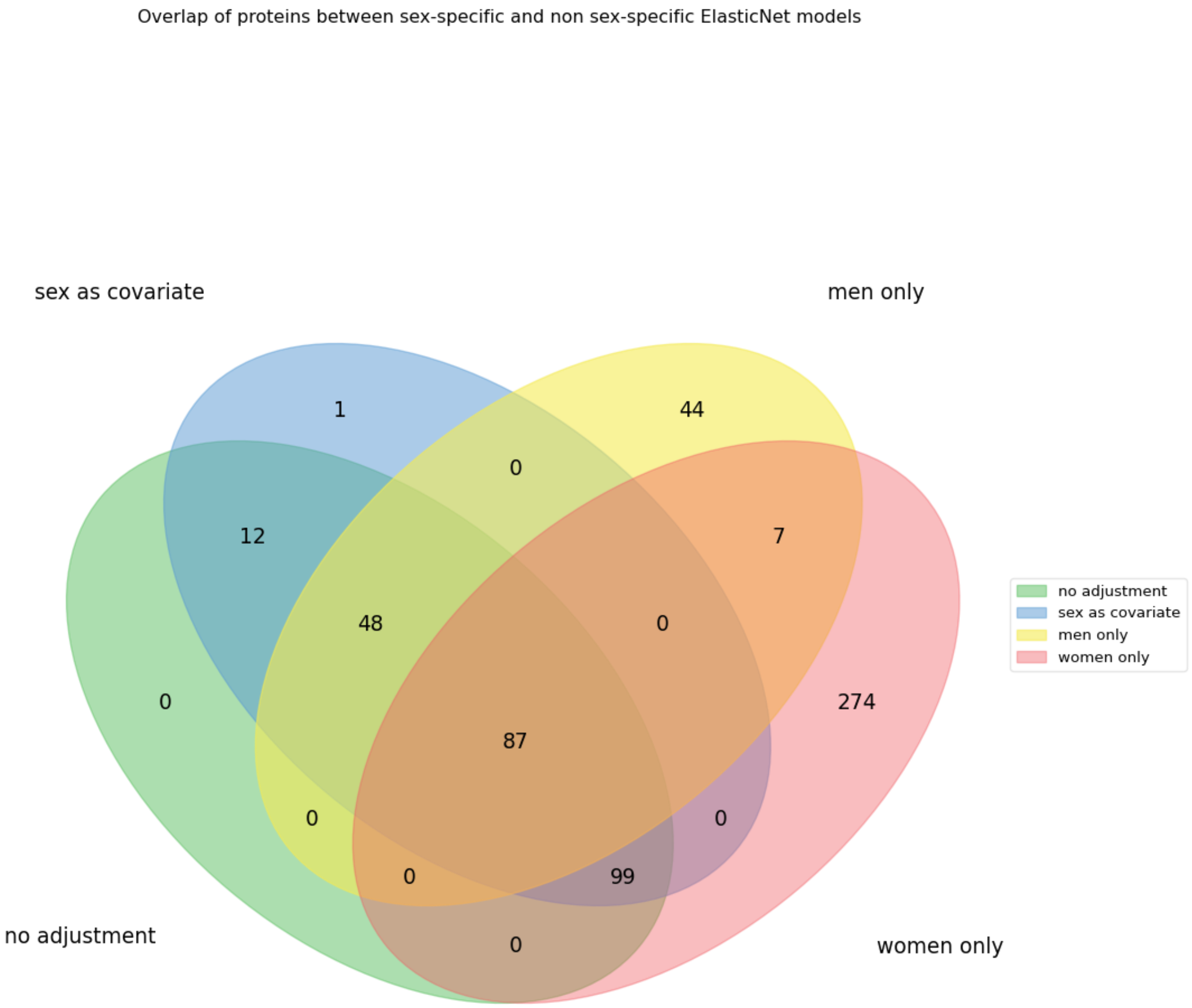

Figure S1. Venn diagram showing the overlap of proteins selected by ElasticNet models for overall mortality across four stratification approaches, related to STAR Methods. ElasticNet models for overall mortality were fitted using four stratification approaches: without sex included, with sex as a covariate, in men only, and in women only. The Venn diagram displays the overlap and unique protein selections obtained from these models.

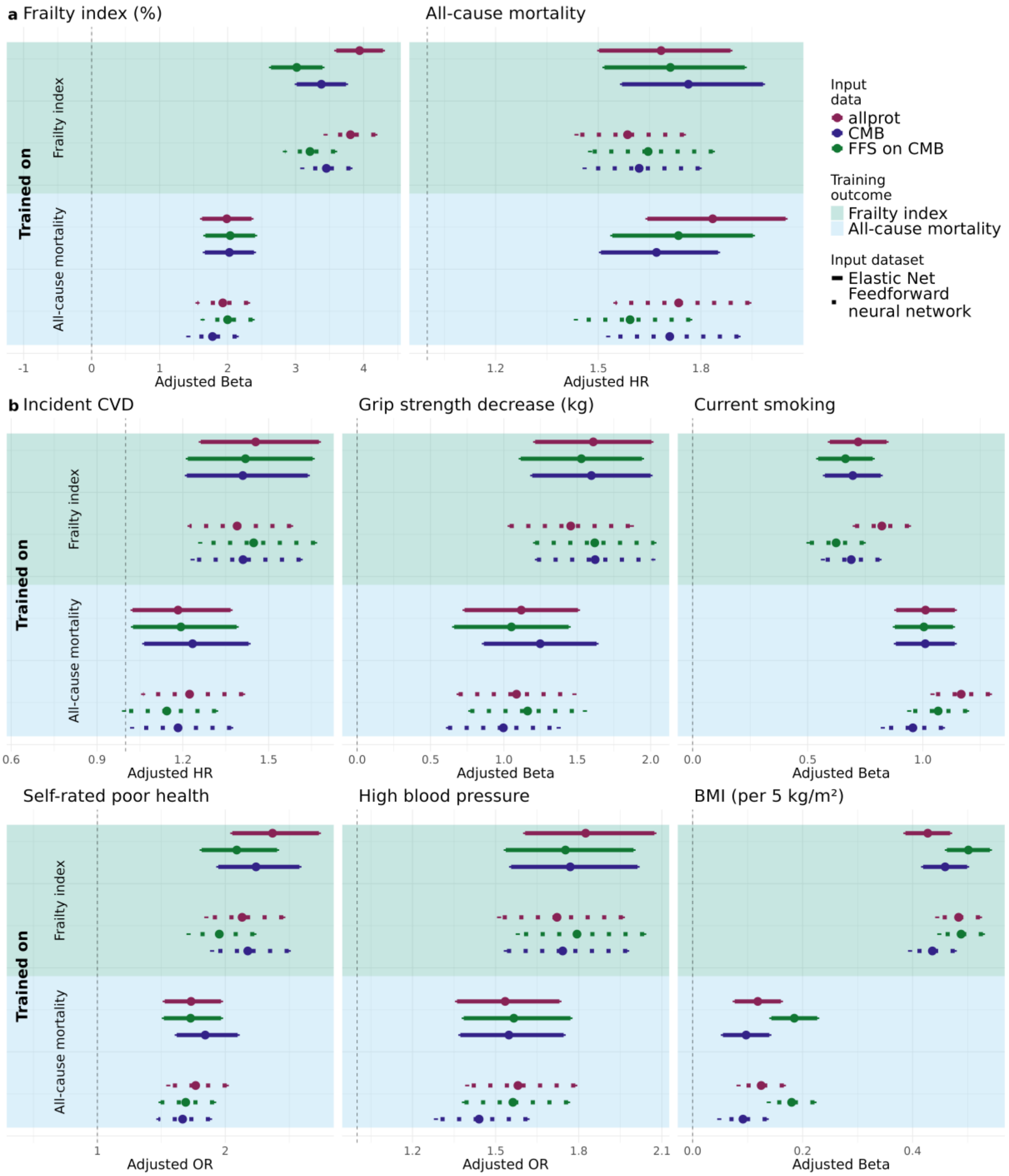

Figure S2. Comparison of biomarkers trained on all proteins, the cardiometabolic panel, and forward feature-selected cardiometabolic proteins for the training endpoints, related to Figures 3 and 4. The figure shows associations of protein-based aging biomarkers trained using the full set of 1,428 proteins from four Olink panels (allprot, red) or the cardiometabolic panel (CMB, blue) or the forward feature selected CMB proteins (FFS) with: A) the training endpoints, namely the frailty index and all-cause mortality, and B) other age-related health indicators. Dots represent the point estimates. Solid and dotted error bars indicate 95% confidence intervals for EN and FNN biomarkers, respectively. The background color indicates the training outcome, with green (upper) for the frailty index and blue (lower) for all-cause mortality. All associations were performed in the independent UKB validation set. See also Table S4.

Allprot indicates the full set of 1428 proteins; CMB the cardiometabolic panel; FFS, forward feature selection; HR, hazard ratio; and OR, odds ratio

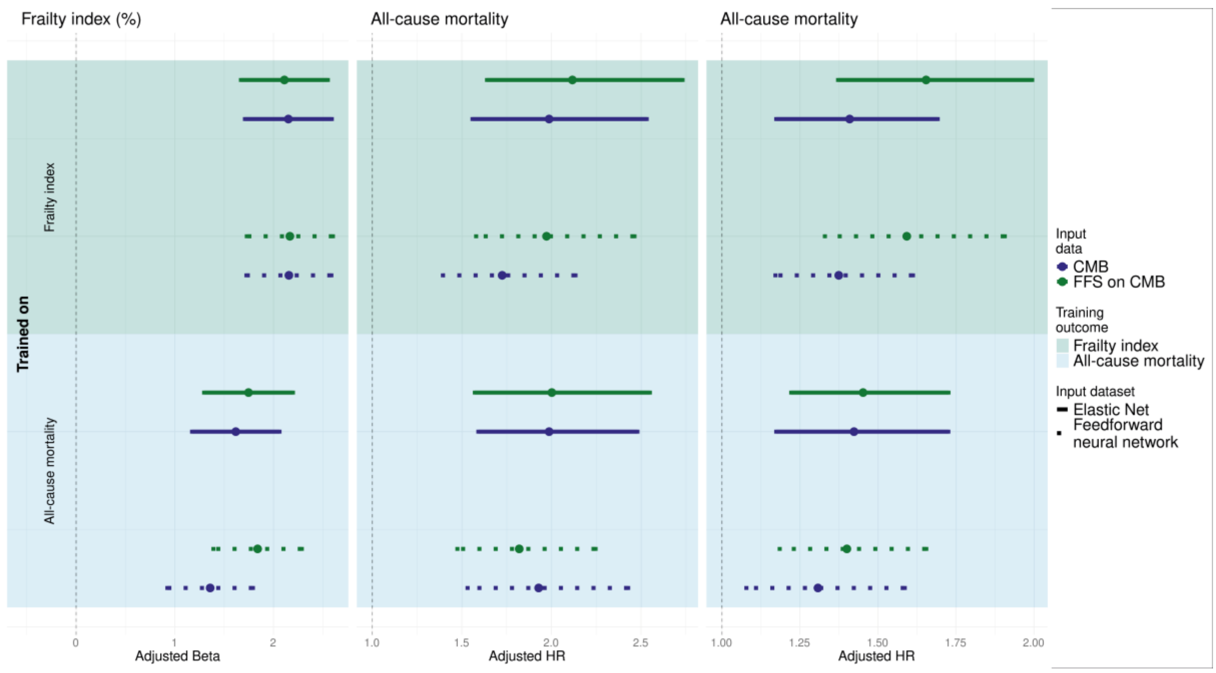

Figure S3. External validation of constructed protein-based aging biomarkers in the Rotterdam Study and the Leiden Longevity Study, related to Figures 4 and 6. The figure shows associations of protein-based aging biomarkers trained using the full set of 1,428 proteins from four Olink panels (allprot, red) or the cardiometabolic panel (CMB, blue) or the forward feature selected CMB proteins (FFS) with: A) the training endpoints, namely the frailty index and all-cause mortality, and B) other age-related health indicators. Dots represent the point estimates. Solid and dotted error bars indicate 95% confidence intervals for EN and FNN biomarkers, respectively. The background color indicates the training outcome, with green (upper) for the frailty index and blue (lower) for all-cause mortality. See also Table S4 and S6.

CMB indicates the cardiometabolic panel; FFS, forward feature selection; and HR, hazard ratio.

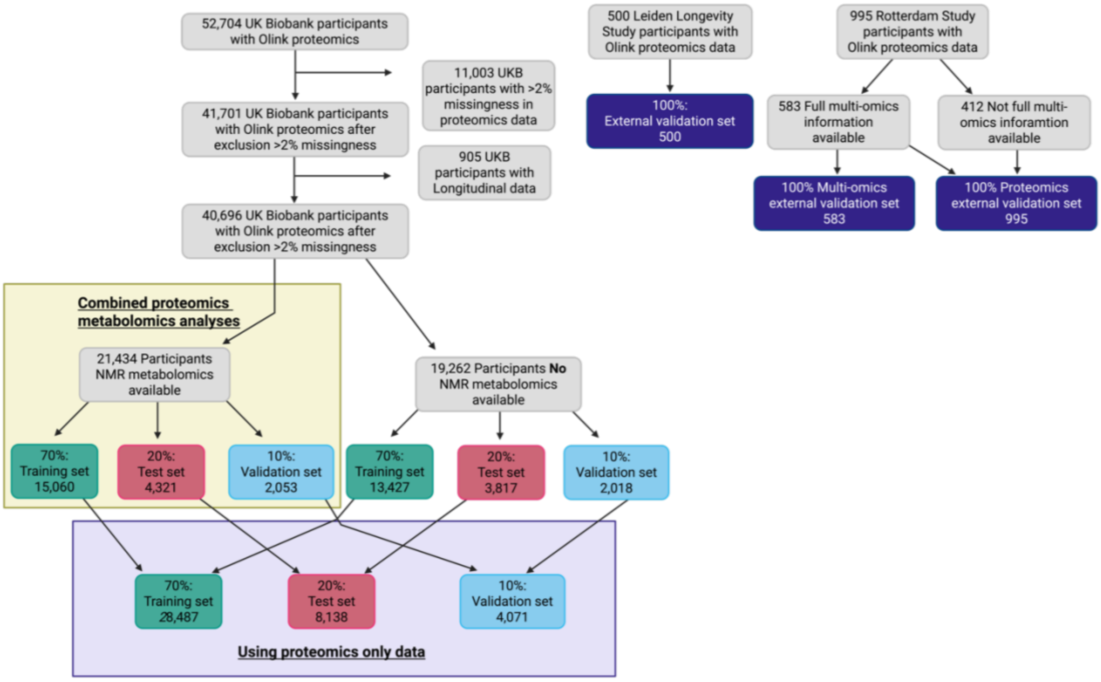

Figure S4. Flowchart of data split creation for the UK Biobank, Leiden Longevity Study, and the Rotterdam Study, related to STAR Methods. This flowchart outlines the creation of training, validation, and external test sets across the UK Biobank, Leiden Longevity Study, and the Rotterdam Study. Participant subsets in the UK Biobank were defined based on availability of proteomics and metabolomics, after which splits were generated for model development and evaluation as detailed in the STAR Methods. The Rotterdam Study dataset consisted of 995 participants with Olink proteomics information with a subset of 583 participants with also information on metabolomics and DNA methylation.

Table S1. The Rockwood frailty index in the UK Biobank, related to STAR Methods.

Type of deficit	Item	Trait	Categories	Coding in Frailty Index
Sensory	1	Glaucoma*	no, yes	Categorized 0/1
	2	Cataracts*	no, yes	Categorized 0/1
	3	Hearing difficulty	no, yes, completely deaf	Categorized 0/1 (combined yes/deaf groups as 1)
Cranial	4	Migraine*	no, yes	Categorized 0/1
	5	Dental problems	ulcers, painful gums, bleeding gums, loose teeth, toothache, dentures	Categorized 0/1 for none vs. any
Mental wellbeing	6	Self-rated health	excellent, good, fair, poor	0 – excellent; 0.25 – good; 0.5 - fair, 1 – poor
	7	Fatigue: frequency of tiredness / lethargy in last two weeks	not at all, several days, more than half, nearly every day	0, 0.25, 0.5, 1, respectively
	8	Sleep: experience of sleeplessness/insomnia	never/rarely, sometimes, usually	Categorized 0, 0.5, 1, respectively
	9	Depressed feelings: frequency in last two weeks	not at all, several days, more than half, nearly every day	0 – not at all, 0.5 – several days, 0.75 -- more than half, 1 – nearly every day
	10	Self-described nervous personality	no, yes	Categorized 0/1
	11	Severe anxiety/ panic attacks*	no, yes	Categorized 0/1
	12	Common to feel loneliness	no, yes	Categorized 0/1
	13	Sense of misery (ever/never)	no, yes	Categorized 0/1
Infirmity	14	Infirmity: long-standing illness or disability	no, yes	Categorized 0/1
	15	Falls in last year	categorical: no falls, one fall, more than one	0, 0.5, 1, respectively
	16	Fractures/broken bones in last five years	no, yes	Categorized 0/1
Cardiometabolic	17	Diabetes*	no, yes	Categorized 0/1
	18	Myocardial infarction*	no, yes	Categorized 0/1
	19	Angina*	no, yes	Categorized 0/1
	20	Stroke*	no, yes	Categorized 0/1
	21	High blood pressure*	no, yes	Categorized 0/1
	22	Hypothyroidism*	no, yes	Categorized 0/1
	23	Deep-vein thrombosis*	no, yes	Categorized 0/1
	24	High cholesterol*	no, yes	Categorized 0/1
Respiratory	25	Breathing: wheeze in last year	no, yes	Categorized 0/1
	26	Pneumonia*	no, yes	Categorized 0/1
	27	Chronic bronchitis/emphysema*	no, yes	Categorized 0/1

	28	Asthma*	no, yes	Categorized 0/1
Musculoskeletal	29	Rheumatoid arthritis*	no, yes	Categorized 0/1
	30	Osteoarthritis*	no, yes	Categorized 0/1
	31	Gout*	no, yes	Categorized 0/1
	32	Osteoporosis*	no, yes	Categorized 0/1
Immunological	33	Hayfever, allergic rhinitis or eczema*	no, yes	Categorized 0/1
	34	Psoriasis*	no, yes	Categorized 0/1
Cancer	35	Any cancer diagnosis*	no, yes	Categorized 0/1
	36	Multiple cancers diagnosed (number reported)	Range from 0 to 6	0 - no cancer or single cancer, 1 - multiple cancers
Pain	37	Chest pain	no, yes	Categorized 0/1
	38	Head and/or neck pain	no, yes (combining responses to pain in head and neck/shoulders)	Categorized 0/1
	39	Back pain	no, yes	Categorized 0/1
	40	Stomach/abdominal pain	no, yes	Categorized 0/1
	41	Hip pain	no, yes	Categorized 0/1
	42	Knee pain	no, yes	Categorized 0/1
	43	Whole-body pain	no, yes	Categorized 0/1
	44	Facial pain	no, yes	Categorized 0/1
	45	Sciatica*	no, yes	Categorized 0/1
Gastrointestinal	46	Gastric reflux*	no, yes	Categorized 0/1
	47	Hiatus hernia*	no, yes	Categorized 0/1
	48	Gall stones*	no, yes	Categorized 0/1
	49	Diverticulitis*	no, yes	Categorized 0/1

* indicates self-reported
The Rockwood frailty index in the UK Biobank, as developed by Williams et al.[1], following the procedure of Searle et al.[2]. The Rockwood frailty index in the UK Biobank consist of 49 items, coded according to the table above. The frailty score is calculated by summing all 49 codes and dividing by the total number of items.

Table S2. The Rockwood frailty index in the Rotterdam Study, related to STAR Methods.

Trait	Item	Trait(s) – additional information	Cutoff value	Based on
Dressing and grooming	1	Able to get clothes from closets or drawers; able to dress; able to shampoo your hair; able to comb your hear or do your make up	Without any difficulty = 0<br>With some difficulty = 0.33<br>With much difficulty = 0.66<br>Unable to do = 1	Stanford Health Assessment Questionnaire
Arising	2	Able to stand up from a straight chair without using your arms for support; able to get in and out of bed	Without any difficulty = 0<br>With some difficulty = 0.33<br>With much difficulty = 0.66<br>Unable to do = 1	Stanford Health Assessment Questionnaire
Eating	3	Able to cut meat and lift a full cup or glass to your mouth; able to open a new carton of milk	Without any difficulty = 0<br>With some difficulty = 0.33<br>With much difficulty = 0.66<br>Unable to do = 1	Stanford Health Assessment Questionnaire
Walking	4	Able to walk outdoors on flat ground; able to climb up five steps	Without any difficulty = 0<br>With some difficulty = 0.33<br>With much difficulty = 0.66<br>Unable to do = 1	Stanford Health Assessment Questionnaire
Hygiene	5	Able to wash and dry your entire body; able to take a shower/bath	Without any difficulty = 0<br>With some difficulty = 0.33<br>With much difficulty = 0.66<br>Unable to do = 1	Stanford Health Assessment Questionnaire
Reach	6	Able to reach and get down a 1kg object from just above your head; able to bend down to pick up clothing from the floor	Without any difficulty = 0<br>With some difficulty = 0.33<br>With much difficulty = 0.66<br>Unable to do = 1	Stanford Health Assessment Questionnaire
Grip	7	Able to open a car door? Able to open jars which have been previously opened	Without any difficulty = 0<br>With some difficulty = 0.33<br>With much difficulty = 0.66<br>Unable to do = 1	Stanford Health Assessment Questionnaire
Riding a bike	8	Able to ride a bike	Without any difficulty = 0<br>With some difficulty = 0.33<br>With much difficulty = 0.66<br>Unable to do = 1	Lawton Instrumental Activities of Daily Living scale
Telephone	9	Able to use the telephone	Without any difficulty = 0<br>With some difficulty or using a customized phone = 0.33<br>With much difficulty = 0.66<br>Unable to do = 1	Lawton Instrumental Activities of Daily Living scale
Meal	10	Able to prepare meals	Without any difficulty = 0<br>With some difficulty = 0.33<br>With much difficulty = 0.66<br>Unable to do = 1	Lawton Instrumental Activities of Daily Living scale
Medication	11	Able to take in medication	Without any difficulty = 0<br>With some difficulty = 0.33<br>With much difficulty = 0.66<br>Unable to do = 1	Lawton Instrumental Activities of Daily Living scale
Laundry	12	Able to do the laundry	Without any difficulty = 0<br>With some difficulty = 0.33<br>With much difficulty = 0.66<br>Unable to do = 1	Lawton Instrumental Activities of Daily Living scale
Financial	13	Able to do finances	Without any difficulty = 0<br>With some difficulty = 0.33<br>With much difficulty = 0.66<br>Unable to do = 1	Lawton Instrumental Activities of Daily Living scale

Depressed affect	14	I felt that I could not shake off the blues even with help from family or friends; I felt depressed; I thought my life had been a failure; I felt lonely; I had crying spells; I felt sad	Rarely or none of the time = 0<br>Some or a little of the time = 0.33<br>Occasionally or a moderate amount of time = 0.66<br>Most or all of the time = 1	The CES-D scale: a self-report depression scale
Positive affect	15	I felt that I was just as good as other people; I felt hopeful about the future; I was happy; I enjoyed life	Rarely or none of the time = 1<br>Some or a little of the time = 0.66<br>Occasionally or a moderate amount of time = 0.33<br>Most or all of the time = 0	The CES-D scale: a self-report depression scale
Somatic and retarded activity	16	I did not feel like eating my appetite was poor; I had trouble keeping my mind on what I was doing felt that everything I did was an effort; I felt fearful; my sleep was restless; I talked less than usual; I could not get “going”	Rarely or none of the time = 0<br>Some or a little of the time = 0.33<br>Occasionally or a moderate amount of time = 0.66<br>Most or all of the time = 1	The CES-D scale: a self-report depression scale
Interpersonal	17	I was bothered by things that usually don’t bother me; people were unfriendly; I felt that people dislike me	Rarely or none of the time = 0<br>Some or a little of the time = 0.33<br>Occasionally or a moderate amount of time = 0.66<br>Most or all of the time = 1	The CES-D scale: a self-report depression scale
Falling	18	How often did you fell the past 12 months?	No falling = 0<br>Less than once a month = 0.5<br>More than once a month = 1	
Joint complaints	19	Did you have joint pain or other complaints from the knees, hips, back or hand?	No = 0<br>Yes = 1	
Forgetfulness	20	Do you sometimes forget what you was about to do?	No = 0<br>Yes = 1	
Aphasia	21	Do you have difficulties with finding the right words?	No = 0<br>Yes = 1	
Liver enzymes	22	ALAS, ALAT, Gamma-glutamyl transpeptidase	All values within the range = 0<br>One or more abnormal values = 1	Serum blood measurement; cutoff values derived from the Laboratory guide Erasmus MC
Creatinine	23		Male 65-115 umol/L = 0<br>Other values = 1<br>Female 55-90 umol/L = 0<br>Other values = 1	Serum blood measurement; cutoff values derived from the Laboratory guide Erasmus MC
Hyperlipidemia	24	High cholesterol or medication against high cholesterol	Statin use and/or cholesterol >6.5 mmol/L<br>No statin use and cholesterol 2.9-6.5 mmol/L	Serum blood measurement; cutoff values derived from the Laboratory guide Erasmus MC
HDL	25		HDL ≥ 1.55 = 0<br>HDL < 1.55 = 1	Serum blood measurement; cutoff values derived from the Laboratory guide Erasmus MC

Systolic blood pressure	26	Measure three times, average is taken	Systolic blood pressure 90-140 = 0<br>Systolic blood pressure 140-160 = 0.5<br>Systolic blood pressure < 90 = 0.5<br>Systolic blood pressure > 160 = 1	Serum blood measurement; cutoff values derived from the Laboratory guide Erasmus MC
MMSE	27	Mini Mental State Examination	Unimpaired >25 = 0<br>Impaired ≤ 25 =1	
LDST	28	Letter-Digit Substitution Test: the number of correct digits	Above mean or less than 1SD below mean = 0<br>One SD below mean = 0.5<br>Two SD below mean = 1	
STROOP	29	Stroop test	Above mean or less than 1SD above mean = 0<br>One SD above mean = 0.5<br>Two SD above mean = 1	
WFT	30	Word Fluency test	Above mean or less than 1SD below mean = 0<br>One SD below mean = 0.5<br>Two SD below mean = 1	
Cancer	31		No = 0<br>Yes = 1	
Lung condition (COPD/Asthma)	32		No = 0<br>Yes = 1	
Cardiovascular diseases	33		No = 0<br>Yes = 1	Prevalent CHD
Stroke	34		No = 0<br>Yes = 1	Prevalent stroke
Diabetes Mellitus	35		No = 0<br>High glucose= 0.5<br>Yes = 1	Prevalent DM and/or glucose levels
BMI	36	Body mass index<br>BMI < 18.5 = underweight<br>BMI ≤25 and ≥18.5 = normal weight<br>BMI <25 and ≤30 = overweight<br>BMI <30 = obese	Normal weight= 0<br>Overweight = 0.5<br>Obese or underweight = 1	
Age-related macular degeneration	37	Fundus photography after pharmacologic mydriasis. The eyes of each participant were graded and classified separately, and the eye with the more severe grade was used to classify the person.	0 = 5-year risk of developing advanced AMD in at least one eye is 0.5%<br>0.25 = 5-year risk is 3%<br>0.50 = 5-year risk is 12%<br>0.75 = 5-year risk is 25%<br>1= 5-year risk is 50%	
Hospital admission	38	Last 12 months	No = 0<br>Yes = 1	

The Rockwood frailty index in the Rotterdam Study, as developed by Schoufour et al.[3], following the procedure of Searle et al.[2]. The Rockwood frailty index in the Rotterdam Study consist of 38 items, coded according to the table above. The frailty score is calculated by summing all 38 codes and dividing by the total number of items.

Table S3. Concordance indexes on the four-protein panel (allprot) dataset using various adjustments for sex, related to STAR Methods.

	Linear	Deep
No adjustment	0.73	0.73
Sex as (input) covariate	0.73	0.73
Men only	0.72	0.70
Women only	0.71	0.72

Table S5. Goodness-of-fit metrics on various multi-omics integration methods, related to STAR Methods.

Dataset / method	ElasticNet		Feedforward Neural Networks	
	Frailty index (R^2)	All-cause mortality (C)	Frailty index (R^2)	All-cause mortality (C)
Using only cardiometabolic proteins	0.24	0.71	0.25	0.71
Cardiometabolic proteins in population subset with metabolomics	0.23	0.69	0.20	0.68
Cardiometabolic proteins and NMR-metabolites concatenated	0.24	0.69	0.23	0.69
Using AJIVE	0.17	0.61	0.14	0.62
PCA then concatenate, using 10 principal components	0.17	0.61	0.16	0.64
PCA then concatenate, using 20 principal components	0.19	0.67	0.17	0.66
Cardiometabolic proteins and MetaboHealth, concatenated	0.23	0.69	0.23	0.68

AJIVE indicates Angle-based Joint and Individual Variation Explained; C, concordance index; NMR, nuclear magnetic resonance; PCA, principal component analysis.

Table S8. Hyperparameters for the ElasticNet models, related to STAR Methods.

	Frailty index		All-cause mortality	
	α	β	α	β
Allprot	2.5×10^{-4}	0.99	1.6×10^{-3}	0.99
CMB	1.5×10^{-4}	0.99	8.4×10^{-3}	0.1
FFS	2.2×10^{-5}	0.99	7.6×10^{-4}	0.69

Allprot indicates, all protein dataset; CMB, cardiometabolic panel; and FFS, proteins resulting after forward features selection

Table S9. Neural network architectures, related to STAR Methods.

	Layer 1	Layer 2	Layer 3	Layer 4
Allprot	Linear: 1428*x 750<br>Dropout: p = 0.2<br>ReLU	Linear: 750 x 400<br>Dropout: p = 0.1<br>ReLU	Linear: 400 x 100<br>Dropout: p = 0.1<br>ReLU	Linear: 100 x 1
CMB	Linear: 344*x 200<br>Dropout: p = 0.2<br>ReLU	Linear: 200 x 100<br>Dropout: 0.1<br>ReLU	Linear: 100 x 10<br>ReLU	Linear: 10 x 1
FFS	Linear: 20-28** x 15<br>ReLU	Linear: 15 x 10<br>ReLU	Linear: 10 x 5<br>ReLU	Linear: 5 x 1

*input dimension is 1 higher when trained on frailty, to give age as an input
**input dimension is 20 for mortality-trained, and 28 for frailty-trained models.

Table S10. Model hyperparameters for the Feedforward Neural Networks, related to STAR Methods.

	Frailty index			All-cause mortality		
	Batch	Learning	γ	Batch	Learning	γ
Allprot	16	5.0×10^{-6}	$5.0 \times 10^{-}$[illegible]	1000	5.0×10^{-6}	$1.0 \times 10^{-}$[illegible]
CMB	22	1.0×10^{-5}	$1.0 \times 10^{-}$[illegible]	2000	5.0×10^{-5}	$5.0 \times 10^{-}$[illegible]
FFS	17	1.0×10^{-4}	$1.0 \times 10^{-}$[illegible]	4060	6.3×10^{-4}	$1.0 \times 10^{-}$[illegible]

Allprot indicates, all protein dataset; CMB, cardiometabolic panel; and FFS, proteins resulting after forward features selection

Supplementary references

1. Williams, D.M., Jylhävä, J., Pedersen, N.L., and Hägg, S. (2019). A Frailty Index for UK Biobank Participants. Journals of Gerontology: Series A *74*, 582–587. https://doi.org/10.1093/gerona/gly094.
2. Searle, S.D., Mitnitski, A., Gahbauer, E.A., Gill, T.M., and Rockwood, K. (2008). A standard procedure for creating a frailty index. BMC Geriatr. *8*, 24. https://doi.org/10.1186/1471-2318-8-24.
3. Schoufour, J.D., Erler, N.S., Kiefte-de Jong, J.C., Kiefte-de Jong, J.C., Voortman, T., Ziere, G., Lindemans, J., Klaver, C.C., Tiemeier, H., Stricker, B., et al. (2017). Design of a frailty index among community living middle-aged and older people: The Rotterdam study. Maturitas *97*, 14–20. https://doi.org/10.1016/j.maturitas.2016.12.002.
