# Supplementary material for: ProtFI, an efficient frailty-trained proteomics-based biomarker of aging, robustly predicts age-related decline
Source: Cell Rep Methods. 2026 Apr 10;6(5):101405. doi: 10.1016/j.crmeth.2026.101405 (PMC13198109; doi:10.1016/j.crmeth.2026.101405)
Supplement: Document S2. Article plus supplemental information [file mmc5.pdf]

Cell Reports
Methods

Article

ProtFI, an efficient frailty-trained proteomics-based biomarker of aging, robustly predicts age-related decline

Graphical abstract

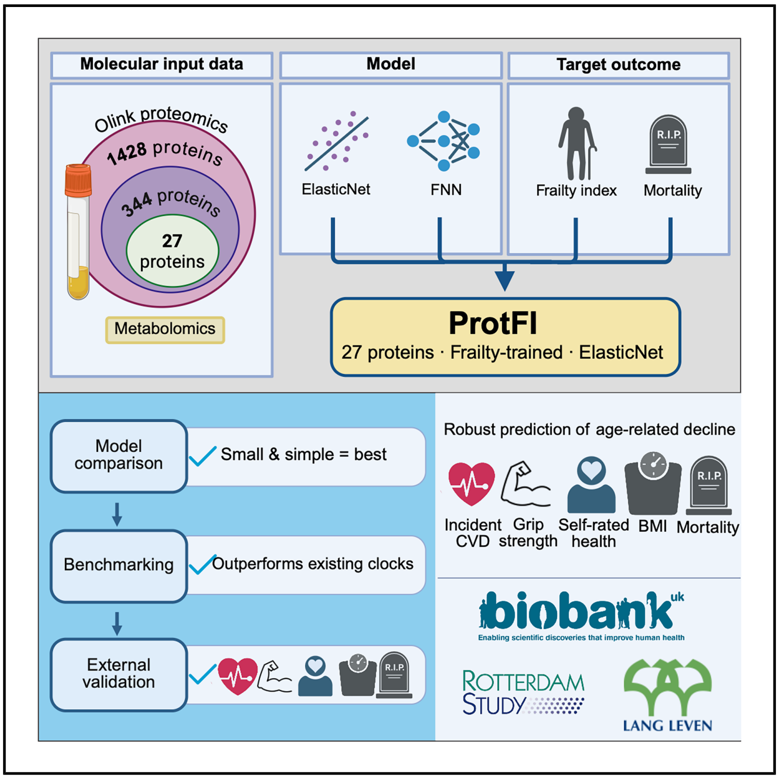

Highlights

- We compare modeling strategies for the development of aging biomarkers
- Simple linear models match or outperform complex neural network models
- We introduce efficient protein biomarkers trained on frailty and mortality
- External validation supports generalizability of ProtFI/ProtMort

Authors

Swier Garst, Lieke Kuiper, Erik van den Akker, ..., Marcel Reinders, P. Eline Slagboom, Joyce van Meurs

Correspondence

m.j.t.reinders@tudelft.nl (M.R.), p.slagboom@lumc.nl (P.E.S.), j.vanmeurs@erasmusmc.nl (J.v.M.)

In brief

Garst et al. systematically compare modeling strategies for aging biomarkers and introduce two efficient protein-based biomarkers trained on frailty and mortality. The findings show that simpler models and minimal protein sets can robustly predict diverse aging outcomes, and that external validation is crucial for ensuring reliable biomarkers.

Garst et al., 2026, Cell Reports Methods 6, 101405
May 18, 2026 © 2026 The Authors. Published by Elsevier Inc.
https://doi.org/10.1016/j.crmeth.2026.101405

CellPress

Cell Reports Methods

CellPress
OPEN ACCESS

Article

ProtFI, an efficient frailty-trained proteomics-based biomarker of aging, robustly predicts age-related decline

Swier Garst,[1,2,10] Lieke Kuiper,[3,4,10] Erik van den Akker,[1,5] Niels van den Berg,[6] Mohsen Ghanbari,[7] Simon Mooijaart,[8] Marian Beekman,[2] Marcel Reinders,[1,11,*] P. Eline Slagboom,[2,11,*] and Joyce van Meurs[3,9,11,12,*]

[1]Delft Bioinformatics Lab, Delft University of Technology, 2628 XE Delft, Zuid-Holland, the Netherlands
[2]Section of Molecular Epidemiology, Department of Biomedical Data Sciences, Leiden University Medical Center, 2333 ZA Leiden, the Netherlands
[3]Department of Internal Medicine, Erasmus MC University Medical Center, 3000 CA Rotterdam, the Netherlands
[4]Center for Prevention, Lifestyle and Health, National Institute for Public Health and Environment (RIVM), 3720 BA Bilthoven, the Netherlands
[5]Leiden Computational Biology Centre, Leiden University Medical Center, 2333 ZA Leiden, the Netherlands
[6]Department of Biomedical Data Sciences, Leiden University Medical Center, 2333 ZA Leiden, the Netherlands
[7]Department of Epidemiology, Erasmus MC University Medical Center, 3000 CA Rotterdam, the Netherlands
[8]Department of Internal Medicine, Division of Gerontology and Geriatrics, Leiden University Medical Center, 2333 ZA Leiden, the Netherlands
[9]Department of Orthopaedics & Sports Medicine, Erasmus Medical Center, 3000 CA Rotterdam, the Netherlands
[10]These authors contributed equally
[11]Senior author
[12]Lead contact
*Correspondence: m.j.t.reinders@tudelft.nl (M.R.), p.slagboom@lumc.nl (P.E.S.), j.vanmeurs@erasmusmc.nl (J.v.M.)
https://doi.org/10.1016/j.crmeth.2026.101405

MOTIVATION Because individuals age at different rates, robust aging biomarkers are needed beyond chronological age. However, the methodological choices that shape such biomarkers have not been compared in a unified framework. This study addresses that gap by evaluating model choice, training targets, and protein selection to derive efficient proteomics-based predictors of frailty and mortality.

SUMMARY

Many molecular aging biomarkers have been developed to capture heterogeneity in individual aging rates. Yet, systematic comparison of the modeling choices underlying these biomarkers has been limited. In this study, we trained aging biomarkers on the Rockwood frailty index (FI) and all-cause mortality using UK Biobank Olink proteomics and metabolomics (^{1}H-NMR) data (n = 40,696). We systematically established the impact of model choice, target outcome, and molecular data source on several age-related outcomes. From this, we developed two aging biomarkers, ProteinFrailty (ProtFI) and ProteinMortality (ProtMort), which are both ElasticNet models that use a minimal set of proteins to predict FI and mortality, respectively. In particular, ProtFI outperformed established aging biomarkers in relation to diverse outcomes, including incident cardiovascular disease, handgrip strength, and self-rated health, both in internal validation and two Dutch external cohorts (n = 995, n = 500). Our findings show that an efficient frailty-trained proteomic biomarker robustly predicts age-related decline.

INTRODUCTION

As individuals age, their risk of functional decline and disease increases.[1,2] However, aging is a heterogeneous process: individuals of the same chronological age may age at different rates and in different ways, resulting in varying risks for physical, cognitive, and functional outcomes.[3] Chronological age is commonly used as a proxy for aging but cannot, by definition, account for individual variation in age-related risk. As a result, the concept of biological age was introduced, aiming to reflect the pace and trajectory of aging on an individual level.[4,5]

Alternatively, the concept of frailty describes a state of increased vulnerability to stressors, resulting from decline across multiple physiological systems.[6] Among these, the Rockwood frailty index (FI), further referred to as the FI, is one of the most widely used frailty measures.[7] Although distinct in concept, quantified frailty scores are frequently used in research and clinical practice as practical proxies for biological age.[5]

© 2026 The Authors. Published by Elsevier Inc.
This is an open access article under the CC BY license (http://creativecommons.org/licenses/by/4.0/).

In addition to clinical assessments such as frailty, molecular or omics biomarkers have more recently been developed to reflect different aspects of biological aging.[4,8] Aging biomarkers are often derived from blood measurements and can span various molecular layers, each reflecting different aspects of the aging process.[4] Compared to the FI, which can include more than 40 age-related deficits,[9,10] blood-based biomarkers are less time consuming to obtain. Since the conception of the Horvath Clock in 2013,[11] numerous epigenetic clocks have been introduced, some of which incorporate clinical markers to enhance predictive accuracy.[12] In parallel, aging biomarkers derived from other *omics* data—including metabolomics,[13] transcriptomics,[14] proteomics,[15] and microbiome profiles[16]—have emerged as alternative approaches to assess biological aging.

Among these, transcriptome and epigenome signatures derived from blood cells are particularly indicative of cellular states that reflect systemic physiological conditions.[12,14] Proteins and metabolite signatures, on the other hand, more directly reflect the functional state of peripheral organs.[17–19]

First-generation omics-based aging biomarkers, known as "aging clocks," were designed to estimate chronological age. In contrast, second-generation aging biomarkers are trained on health-related metrics, such as composite scores of disease burden or mortality risk.[13,20] Studies have demonstrated that biomarkers trained on mortality and age-related health data outperform those solely trained on chronological age in predicting health outcomes.[21,22] Given the broad spectrum of physiological impairments captured by the FI, we hypothesize that a proteomics-based aging biomarker trained to predict frailty could provide a robust and mechanistically relevant tool for assessing biological aging. Recently, using frailty as a training outcome has received considerable attention. Some works focus on the use of alternative measures to estimate frailty itself, but do not evaluate frailty as a training target for aging biomarkers.[23–25] Other studies have attempted developing biomarkers using frailty, but either lack analysis regarding associations beyond frailty itself,[26] or are missing external validation of these associations.[26,27]

More recently, advances in machine learning have transformed aging biomarker development by enabling more complex, multi-variate approaches. Despite methodological advancements in aging biomarker development, practical implementations of aging biomarkers vary widely across the field. More critically, the choice for using more complex models is often undermotivated, lacking comparison to simpler model baselines, while challenges using these more complex models persist. Systematic work exploring key steps in biomarker development, such as model choice and training target selection, is lacking. Therefore, such choices in existing biomarker work remain mostly underscrutinized.

Furthermore, challenges remain in validation and standardization. Many new biomarkers lack rigorous external replication, and are rarely benchmarked against established biomarkers, making it difficult to assess their relative effectiveness. Consequently, a growing array of biomarkers based on single or multi *omic*-data has emerged,[4,8] but without systematic comparisons, their clinical relevance and reliability remain uncertain.

In this study, we developed aging biomarkers based on using Olink proteomics and Nightingale Health (^{1}H-NMR) metabolomics data. We assessed the impact of model choice, target outcome, and molecular data source, identifying key factors that influence biomarker performance. Based on this analysis, we introduce two aging biomarkers: ProteinFrailty (ProtFI) and Protein Mortality (ProtMort), which leverage a minimal subset of proteins to predict frailty and mortality, respectively. By comparing ProtFI and ProtMort to previously established aging biomarkers, we demonstrate that an efficient protein-based approach, trained to predict frailty, provides robust and generalizable insights into aging phenotypes. By validating both aging biomarkers in two independent cohorts, we underscore the need to establish biomarker robustness across independent cohorts and populations.

RESULTS

Study outline

We systematically investigated the use of proteomics for developing aging biomarkers. We evaluated key design choices in this process, structured into three main components: (1) model selection, (2) protein selection, and (3) prediction target. To assess model selection, we constructed aging biomarkers using ElasticNet (EN) models and deep feedforward neural networks (FNNs). These models were selected because they represent popular choices within two categories of models, being simple but linear (EN), or complex and non-linear (FNN). These models were trained on various subsets of plasma proteins from the UK Biobank (UKB),[28,29] ranging from four Olink panels (1,428 proteins), to the cardiometabolic (CMB) panel only (344 proteins), to a subset of the CMB panel (20/27 proteins). Each biomarker was designed to predict either the FI (determined at the same time point as the protein measurement) or all-cause mortality. In the UKB, the FI consisted of 49 categorical deficits, which are categorized into sensory, cranial, mental well-being, infirmity, CMB, respiratory, musculoskeletal, immunological, cancer, pain and gastrointestinal related deficits[9,10] (specific FI deficits used in this study are detailed in STAR Methods and Tables S1 and S2).

All aging biomarkers were trained, tested, and internally and independently validated on the UKB data, and externally validated in the Rotterdam Study (RS)[30] and the Leiden Longevity Study (LLS).[31] Table 1 summarizes the population characteristics. The UKB actively recruited National Health Service (NHS) patients[28] and exhibits a strong healthy volunteer bias.[32] In contrast, the RS is a population-based study,[30] while the LLS comprises offspring of long-lived individuals and their partners.[31] For further details, see the experimental model and study participant details section in the STAR Methods. Figure 1 shows a graphical representation of the study outline. For a selected set of aging biomarkers, we conducted association analysis with a range of age-related health outcomes, chosen based on their availability with statistical power across the three cohorts. Initial analyses on sex-specific modeling showed sizable overlap in protein selection for the EN model on the full 1,428 protein set, with the exception for the model trained on only female participants (Figure S1). Furthermore, stratification showed no improvement in model performance (Table S3). We therefore decided not to stratify by sex. All reported effect estimates in the UKB refer to those obtained from the internal validation set.

Table 1. Population characteristics

	UK Biobank	Rotterdam Study	Leiden Longevity Study
Number of participants	40,696	995	500
Age in years	57.5 (8.2)	62.7 (5.8)	66.0 (6.7)
Number of women	21,941 (53.9)	568 (57.1)	252 (50.4)
Frailty index (0–1)	0.13 (0.07)	0.13 (0.07)	–
Died during follow-up	4,497 (11.1)	68 (6.7)	95 (19.0)
Years of follow-up for all-cause mortality	13.7 [13.0, 13.5]	10.3 [9.6, 10.7]	13.8 [13.4, 14.1]
Highest attained education			
Primary education	7,293 (17.9)	68 (6.8)	17 (3.4)
Lower vocational or intermediate general education	11,079 (27.2)	323 (32.5)	119 (23.8)
Intermediate vocational or secondary education	8,844 (21.7)	280 (28.1)	190 (38.0)
Higher vocational education or university	12,764 (31.4)	321 (32.3)	168 (33.6)
BMI in kg/m^2	27.5 (4.8)	27.3 (4.4)	25.5 (3.7)
Current smoker	4,357 (10.7)	133 (13.4)	31 (6.2)
Maximum handgrip strength in kilogram	32.5 (11.3)	31.2 (10.3)	40.3 (11.7)
Self-rated health poor	11,058 (27.2)	85 (8.5)	49 (9.8)
High blood pressure	11,563 (28.4)	570 (57.3)	119 (23.8)
Prevalent cardiovascular disease	2,040 (5.0)	83 (8.3)	31 (6.2)
Incident cardiovascular disease	3,587 (8.8)	60 (6.6)	28 (7.3)
Years of follow-up for incident cardiovascular disease	11.7 [11.0, 12.5]	6.5 [6.0, 6.9]	5.0 [4.4, 5.9]
Percentage of lymphocytes	28.8 (7.7)	35.0 (7.8)	–
Percentage of monocytes	7.1 (2.6)	6.4 (2.0)	–
Part of multi-omics set	21,434 (52.7)	583 (58.6)	500 (100)
Selected as PPP-participant	4,898 (12.0)	–	–

Values represent mean and SD between parentheses for continuous variables (chronological age, FI, BMI, maximum handgrip strength, percentage of lymphocytes, and percentage of monocytes), median, and the interquartile range between brackets for both the mortality as well as the incident cardiovascular disease follow-up time, and the number of participants and the percentage between brackets for the categorical variables (number of women, died during follow-up, highest attained education, current smoker, prevalent cardiovascular disease, incident cardiovascular disease, being part of the multi-omics set, and being non-randomly selected by the UK Biobank Pharma Proteomics Project [PPP] as participant).

Comparison between linear and deep learning models shows no benefit for more complex models

We first evaluated the impact of model complexity on aging biomarker performance. We started by using the set of four Olink panels, together making up 1,428 proteins (allprot), and trained aging biomarkers based on outcomes and modeling approach. Markers were trained to either predict all-cause mortality or the FI, using two approaches: a linear EN model and a FNN. This resulted in four biomarkers: EN trained on the FI and mortality (EN-allprot-FI and EN-allprot-mort) and FNN trained on the FI and mortality (FNN-allprot-FI and FNN-allprot-mort).

To assess model performance, we employed bootstrapping with replacement, generating 100 resampled training sets. For each resampled set, we applied the predetermined hyperparameters and evaluated performance metrics on the independent validation set, including R^2 score for the FI as outcome and the concordance index (C-index) for mortality as outcome. We then compared the distribution of these performance metrics between the resulting EN and FNN aging biomarkers. Bootstrapping revealed a significant difference in performance (p value after false discovery rate adjustment [pFDR] < 0.001), favoring the EN model over the FNN model for both the FI and mortality (Figure 2). On average, the EN biomarkers exhibited an R^2 that was 0.034 (standard deviation [SD] 0.008) higher and a C-index that was 0.015 (SD 0.010) higher than their FNN counterparts. These findings suggest that increased model complexity, as in FNNs, does not enhance performance.

Associations with non-training endpoints remain similar when reducing the number of proteins

For theoretical and future practical reasons, we asked the question how much redundancy exists in the information of the full set of proteins used so far. Therefore, we evaluated the impact of reducing the number of proteins used for aging biomarker training, decreasing the input from 1,428 to the CMB panel of 344 proteins (based on Olink panel distribution, STAR Methods for details). This selection was guided by the availability of this panel in the RS and LLS cohorts, enabling external validation.

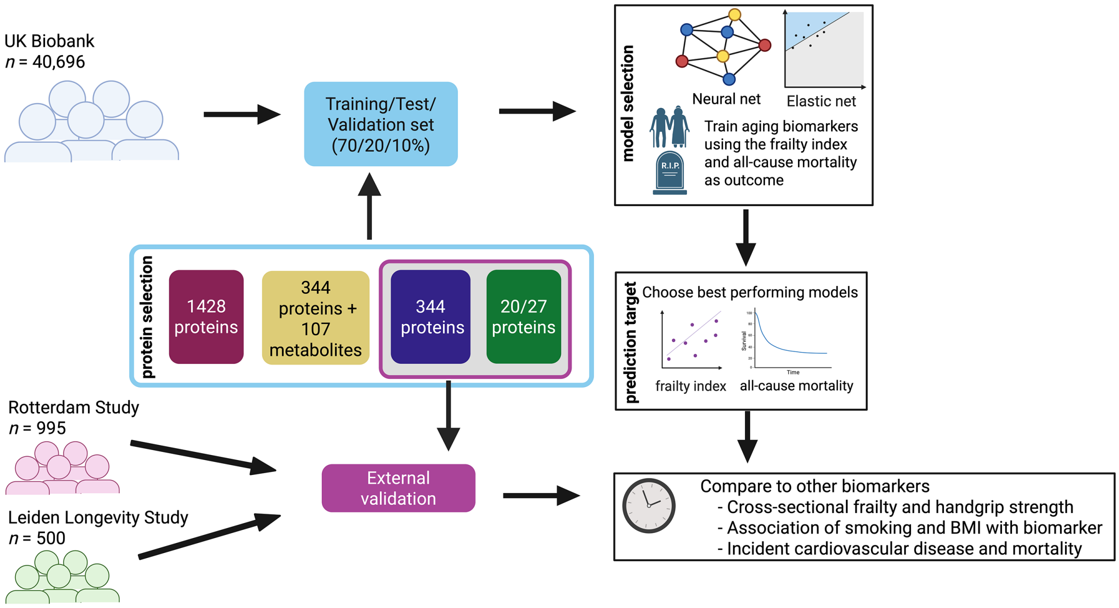

Figure 1. Overview of the study design

We used proteomics data from the UK Biobank as training data for all biomarkers. By creating a 70/20/10 training/test/validation split, we investigated the effect of the number of proteins included in a biomarker, as well as the effect of including metabolomics data. Furthermore, we compared linear EN and non-linear FNN models to investigate the effect of using more complex models for biomarker training. We trained all our models to either predict the FI or mortality, in order to investigate the effect of training outcome. Finally, biomarkers based on the two smallest protein subsets were externally validated in two cohorts, the RS and the LLS (as well as the validation set in the UK Biobank), by means of association analyses on several aging-related phenotypes.

As in the previous analysis, we trained aging biomarkers using EN and FNN models on the UKB training set to predict either the FI or all-cause mortality, resulting in four CMB-based biomarkers: EN-CMB-FI, EN-CMB-mort, FNN-CMB-FI, and FNN-CMB-mort. The performance metrics (R^2 score or C-index) of the resulting CMB-based aging biomarkers on the independent UKB validation set were slightly, yet significantly, lower compared to the aging biomarkers trained on the full set of proteins (Figures 2C and 2D; pFDR < 0.001). Specifically, the EN aging biomarker trained on all proteins (EN allprot) demonstrated, on average, a 0.047 (SD 0.003) higher R^2 for the FI prediction and a 0.015 (SD 0.005) higher C-index for all-cause mortality prediction compared to the EN aging biomarker trained on the CMB panel (EN CMB).

In the UKB validation set, we then examined the associations per SD increase in the age-accelerated biomarkers, which reflects the component of the biomarker independent of chronological age and indicates whether individuals are biologically older or younger than the population average, trained on either the allprot or CMB protein set with various age-related health indicators (see STAR Methods, section "association analysis for details," all results in Table S4). These analyses were adjusted for sex, socio-economic status, smoking status, BMI, and cell counts. Figure 3 illustrates the associations between the aging biomarkers and their respective training targets: the FI and all-cause mortality. As expected, aging biomarkers generally exhibited stronger associations with the specific health indicator they were trained to predict—the FI for FI-trained biomarkers (top left, Figure 3) and mortality for mortality-trained biomarkers (bottom right, Figure 3). However, none of these differences were statistically significant after FDR correction (pFDR > 0.10).

For FI-trained biomarkers, the adjusted percentage increase in the FI per SD in the aging biomarker was 3.94 (95% confidence interval [CI]: (3.60, 4.28)) for EN-allprot-FI and 3.38 (95% CI: (3.02, 3.74)) for EN-CMB-FI; the FNN-based aging biomarkers exhibited similar associations.

Similarly, mortality-trained biomarkers demonstrated slightly lower but comparable associations with mortality. The EN-allprot-mort biomarker was associated with a hazard ratio (HR) of 1.83 (95% CI: (1.64, 2.05)), while EN-CMB-mort had an HR of 1.67 (95% CI: (1.51, 1.85)). The FNN-based biomarkers followed a similar trend.

Notably, this pattern was not observed for the alternative training outcome (i.e., frailty when predicting mortality and vice versa; top right and bottom left, Figure 3A, left image), where differences between aging biomarkers trained on allprot or CMB were even smaller (pFDR > 0.99). When assessing associations with all-cause mortality, FI-trained biomarkers demonstrated equivalent performance when comparing the allprot and CMB protein sets. The EN-allprot-FI aging biomarker yielded an HR of 1.68 (95% CI: (1.50, 1.89)), while the EN-CMB-FI biomarker had an HR of 1.76 (95% CI: (1.57, 1.98)). The FNN-based aging biomarkers exhibited slightly lower HRs, though still comparable between the two protein sets.

The same held true in the reverse case (Figure 3A, right image), where mortality-trained biomarkers were evaluated against the FI. Both EN- and FNN-based biomarkers showed similar performances across protein sets; the EN-allprot-mort biomarker showed an adjusted percentage increase of 1.99 (95% CI: (1.62, 2.35)), while the EN-CMB-mort biomarker had an increase of 2.03 (95% CI: (1.66, 2.39)). Altogether, this suggests that,

CellPress
OPEN ACCESS

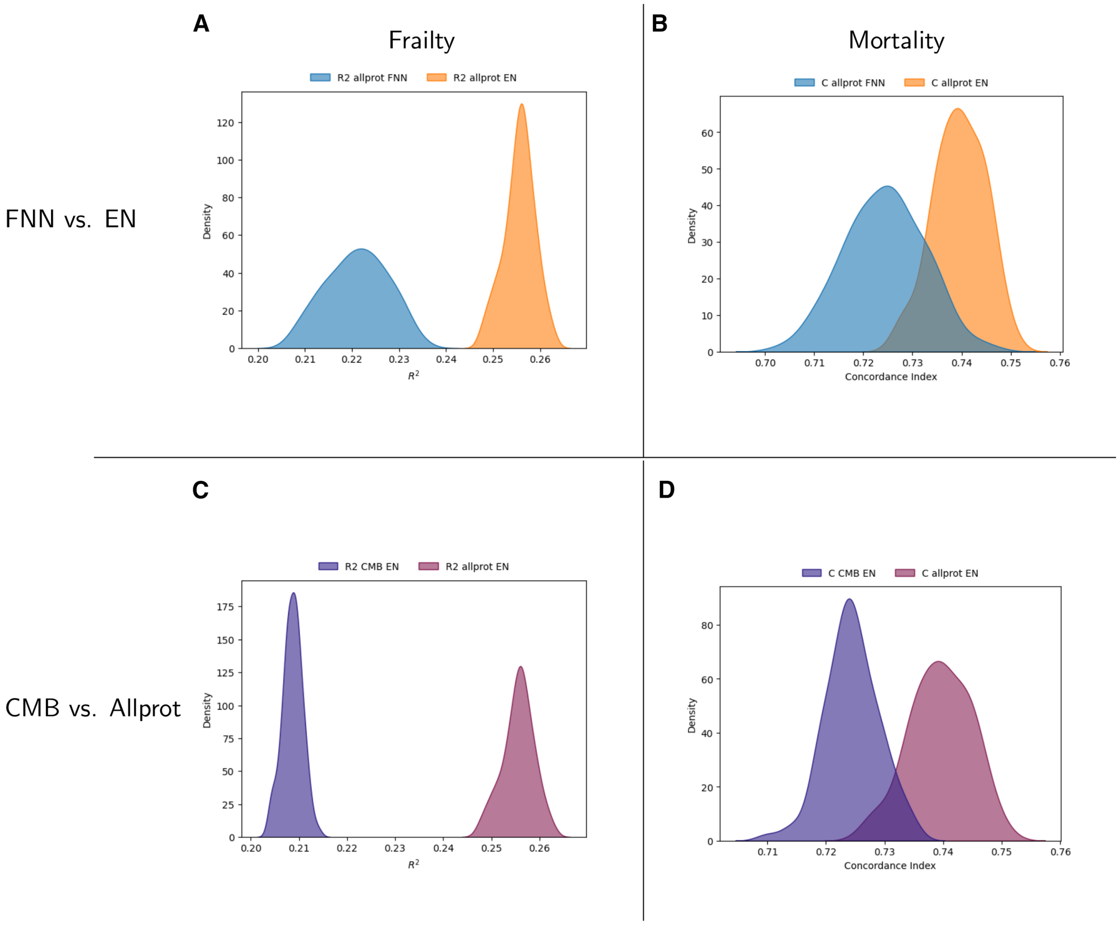

Figure 2. Density plots

Density plot showing (A and C) R^2 score (when trained on frailty) and (B and D) C-index (when trained on mortality) for aging biomarkers, comparing EN (orange) and FNN (blue) in (A and B), and the allprot (purple) and CMB (red) protein sets when training EN in (C) and (D). Distributions reflect performance on the fixed 10% independent validation set across models trained with the pre-selected hyperparameters on 100 bootstrap resamples of the combined training and test set (90% of the original UKB data on which the models were optimized).

although using more proteins increases performance on the training target, it does not benefit associations with other age-related health indicators.

Subsequently, we evaluated the impact of data source on biomarker performance. We investigated the integration of Olink proteomics data (CMB panel only) with 107 non-derived Nightingale Health (^{1}H-NMR) metabolite markers using three approaches: (1) direct concatenation of proteins and metabolites, (2) applying principal component analysis (PCA) separately to each data type followed by concatenation of the first 10 or 20 principal components of each modality, and (3) AJIVE, which finds a common subspace between the two data modalities (methods detailed in STAR Methods, section “multi-omics”). However, none of the integration methods, whether using FNN or EN, resulted in higher aging biomarkers’ performance metrics (R^2 or C-index); see Table S5.

Associations with other health outcomes

Next, we investigated to what extend the FI or mortality trained markers predicted relevant age-related health indicators. Figure 3B demonstrates that a model trained on the FI predicts these health outcomes better than a model trained on mortality, with the exception of smoking. Second, regardless of the training method, biomarkers derived from the reduced set of proteins yield comparable associations (pFDR > 0.14).

Furthermore, for some health indicators, such as the association between FI-trained FNN biomarkers and self-rated poor health, the FNN-CMB biomarker showed slightly higher point estimates (odds ratio [OR] = 2.13, 95% CI: (1.85, 2.45) for FNN-allprot-FI vs. OR = 2.18, 95% CI: (1.90, 2.50) for FNN-CMB-FI]. Similarly, for incident cardiovascular disease, the FNN-CMB-FI biomarker yielded higher point estimates. This indicates that this subset of proteins is able to retain important information with respect to broader aging factors.

Forward feature selection is an effective method in selecting highly associative proteins for an aging biomarker

Biological age markers ideally capture a range of aging-related health outcomes to indicate overall health of individuals with rather a minimum than a maximum of molecular features. We

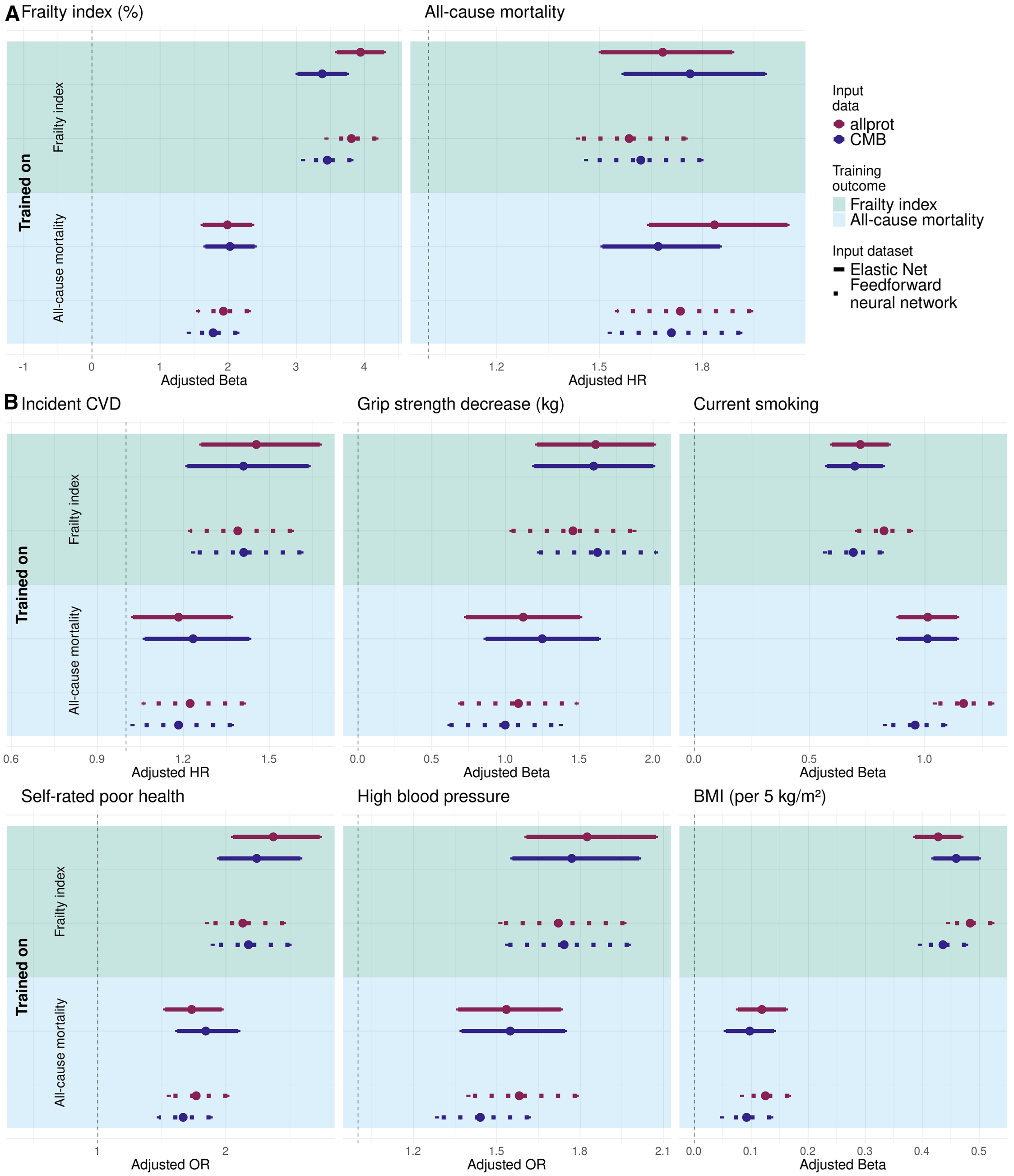

Figure 3. Health indicator associations of aging biomarkers trained on allprot and CMB proteins on the UKB

The figure shows associations of protein-based aging biomarkers trained using four Olink panels (allprot, red) or only the CMB panel (blue) with: (A) the training endpoints, namely the FI and all-cause mortality, whereas (B) other age-related health indicators. Dots represent the point estimates. Solid and dotted error bars indicate 95% CIs for EN and FNN biomarkers, respectively. The background color indicates the training outcome, with green (upper) for the FI and blue (lower) for all-cause mortality. All performance metrics were measured in the independent UKB validation set. See also Table S4.

observed that the subset of CMB proteins as compared to allprot can retain equivalent associations with a range of health outcomes beyond those they were trained on. Therefore, we aimed to identify a "minimal selection" of proteins that maintain robust associations with various age-related health indicators tested earlier. To achieve this, we applied a forward feature selection (FFS) approach to identify proteins from the CMB protein set that incrementally increase goodness-of-fit metrics by more than 0.001, i.e., R^2 in the case of the FI (27 proteins plus chronological age) or the C-index in the case of all-cause mortality (20 proteins) in the UKB training set (see STAR Methods for details). The final protein lists for ProtFI and ProtMort are shown in Table 2. Of these proteins, only the MET protein has been discussed in earlier GWAS studies.[33–36] In total, only six proteins

Table 2. The proteins selected in the FFS procedure, together with the accompanying beta values of the linear regressor used for selection

Frailty index		All-cause mortality	
Protein name	Beta value	Protein name	Beta value
ACE2	0.00566	BOC	0.147
ADGRE5	−0.00634	BPIFB1	0.142
ALCAM	0.00706	CCL27	−0.093
ART3	−0.00689	CHRDL2	0.120
CHRDL2	−0.00575	CNTN1	−0.077
COL6A3	0.00412	COL1A1	−0.111
CTSB	−0.00354	COL6A3	0.284
CTSL	0.00321	CRTAC1	−0.127
EGFR	−0.00501	DCN	−0.101
ENPP2	−0.00411	DLK1	−0.151
FAP	−0.00531	DPT	−0.095
FETUB	0.00366	GDF15	0.256
GDF15	0.00876	ICAM1	0.115
IGFBP3	−0.00430	ITGB2	−0.156
LEP	0.00927	KITLG	−0.096
LGALS3	0.00528	PCSK9	0.097
LRP11	0.00378	REN	0.090
LTBP2	0.00506	SPARCL1	−0.084
MET	−0.00345	SPP1	0.211
NRP1	0.00366	XG	−0.091
PCSK9	0.00410	N/A	N/A
PLA2G1B	−0.00297	N/A	N/A
PLTP	−0.00354	N/A	N/A
REN	0.00611	N/A	N/A
SOST	−0.00348	N/A	N/A
TNFSF13B	0.00477	N/A	N/A
TSHB	−0.00409	N/A	N/A
Chronological age	−0.00486	N/A	N/A

from the full CMB panel were overlapping with loci found in these GWAS studies.

Using these minimal protein sets, we created aging biomarkers using EN, named ProtFI and ProtMort, predicting the FI and all-cause mortality, respectively. For comparative reasons, we also trained FNN models on the same reduced protein sets to predict frailty (FNN-FFS-FI) and mortality (FNN-FFS-mort). We assessed the associations of the resulting FFS aging biomarkers with the FI, all-cause mortality, and various health indicators in the independent UKB validation set.

Reducing the number of proteins from 344 to 27 for FI-based models and to 20 for all-cause mortality models, resulted in some general attenuation of associations with the respective training outcomes (Figure 4A). However, these differences were not statistically significant for any aging biomarker (all pFDR > 0.86). When restricting the analysis to 27 proteins and age, the adjusted effect size (β) on the FI itself for the FI-trained EN biomarker decreased from 3.38 (95% CI: (3.02, 3.74)) for EN-CMB-FI to 3.02 (95% CI: (2.64, 3.39)) for ProtFI. A similar attenuation was observed for the FNN-based aging biomarkers.

For all-cause mortality-trained biomarkers, the HR for mortality prediction for ProtMort was slightly higher than for the EN-CMB-mort estimate (ProtMort: HR = 1.73, 95% CI: (1.54, 1.95) vs. EN-CMB-mort: HR = 1.67, 95% CI: (1.51, 1.85)), though this difference was not statistically significant. In contrast, for mortality-trained FNN biomarkers, a greater number of proteins resulted in a stronger association with the training outcome. This suggests that, overall, using a larger protein set enhances predictive performance for the training outcome, consistent with findings presented in Figure 3.

Figure 4B further compares the associations of FFS and CMB aging biomarkers with various health outcomes. The full comparison between allprot-, CMB-, and FFS-based biomarkers can be found in Figure S2. Despite being trained on fewer than one-tenth of the proteins used in the CMB aging biomarkers, both EN- and FNN-trained FFS aging biomarkers showed no significant attenuation in their associations with health outcomes compared to CMB-trained biomarkers within the same modeling framework and training outcome (all pFDR > 0.71). In some cases, the point estimates for the FFS aging biomarkers were even higher than those for the CMB aging biomarkers, such as for the association with incident cardiovascular disease in FI-trained aging biomarkers (HR = 1.45, 95% CI: (1.26, 1.66) for FNN-FFS-FI vs. HR = 1.41, 95% CI: (1.23, 1.61) for FNN-CMB-FI) and prevalent high blood pressure in mortality-trained aging biomarkers (OR = 1.56, 95% CI: (1.39, 1.76) for FNN-FFS-mort vs. OR = 1.44, 95% CI: (1.28, 1.62) for FNN-CMB-mort). These findings further suggest that protein-based aging biomarkers trained on a reduced protein set maintain their ability to capture key aspects of the aging process, despite the substantial reduction in the number of proteins. Furthermore, in a post hoc sensitivity analysis adjusting ProtFI for cross-sectional FI, the association with all-cause mortality remained stable (HR = 1.47, 95% CI: (1.28, 1.69)).

Comparison with published aging biomarkers

Given that biomarkers developed using less complex models with a reduced number of proteins demonstrated robust associations with aging phenotypes, we proceeded with the EN-trained FFS aging biomarkers, ProtFI (FI-trained) and ProtMort (mortality-trained). We then compared ProtFI and ProtMort with previously published aging biomarkers (see STAR Methods for details). Specifically, we benchmarked ProtFI and ProtMort against the proteomics-based aging biomarker ProteinAge and ProteinAge20, trained on chronological age,[37] and two mortality-trained biomarkers, the ProteinMortality score,[38] and the proteomic aging clock (PAC).[39] Additionally, we included two metabolomics-based aging biomarkers, both of which predict mortality: the Metabo Mortality score[38] and MetaboHealth,[13] which—unlike the other biomarkers—was not trained in the UKB. Since previous studies identified GDF15 as a key protein associated with mortality,[19] and given that it was the most important protein selected by our FFS procedures for both the FI and all-cause mortality, we also evaluated GDF15 alone.

Figure 5 demonstrates that, despite using fewer proteins than PAC (128 proteins, derived from 2,923 proteins), the ProteinMortality score (201 proteins) or ProteinAge (204 proteins), ProtFI exhibited the strongest associations with the FI, incident cardiovascular disease, self-rated poor health,

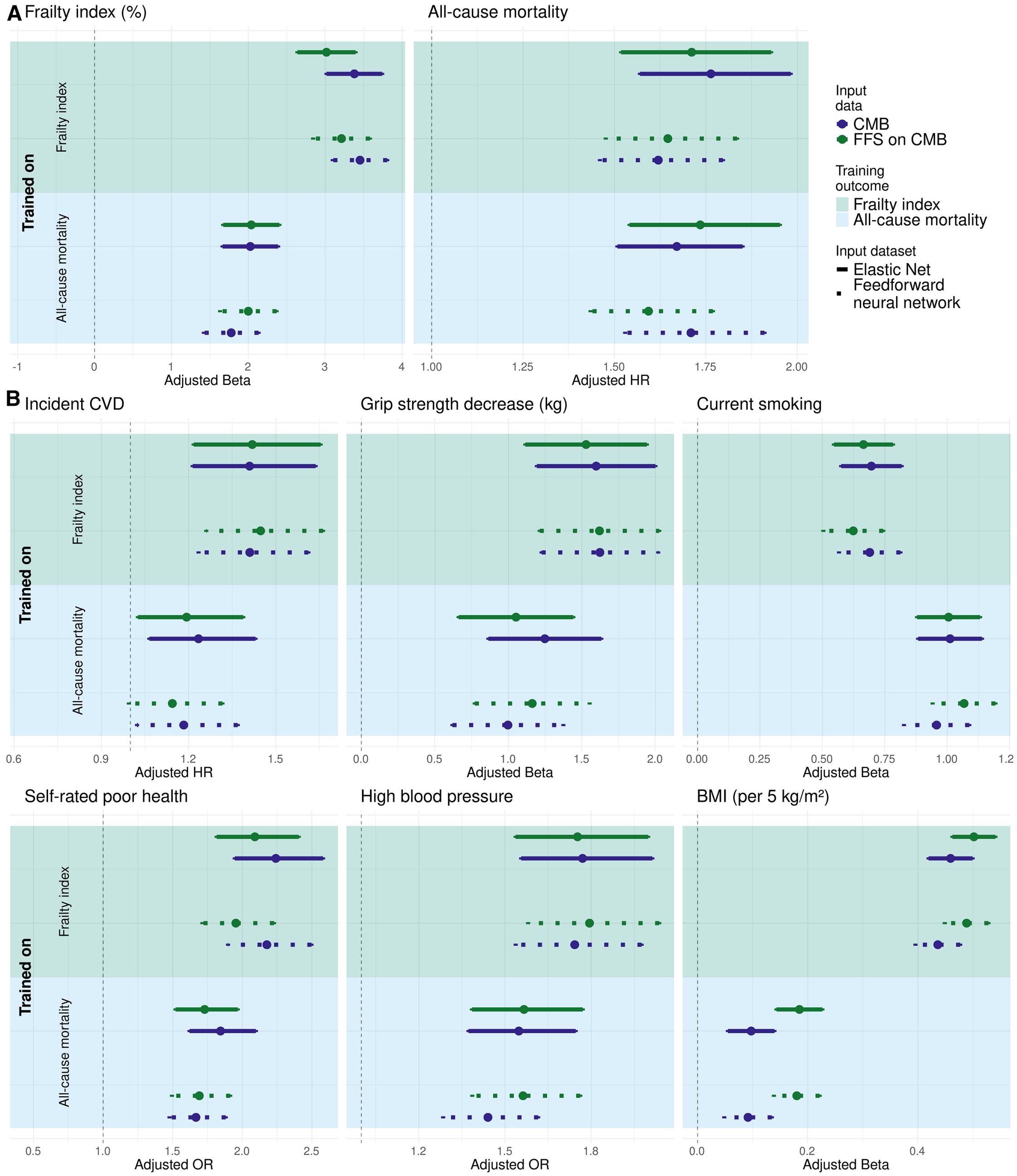

Figure 4. Health indicator associations of aging biomarkers trained on CMB and FFS-selected proteins
The figure shows associations of protein-based aging biomarkers trained using the CMB panel or the forward feature selected CMB proteins (FFS) with: (A) the training endpoints, namely the FI and all-cause mortality and (B) other age-related health indicators. See Figure 3 for figure elaboration. All performance metrics were measured in the independent UKB validation set. See also Table S4.

prevalent high blood pressure, and was most prominently associated with BMI. The differences in association between ProtFI and the next most associative model (per endpoint) remain significant for the FI and the association with BMI. While stronger associations for ProtFI might be expected for indicators included in the FI (e.g., high blood pressure and self-rated poor health), similar trends were observed for independent measures like incident cardiovascular disease and handgrip strength.

In contrast, for all-cause mortality, PAC showed the strongest association (ProtFI: HR = 1.71, 95% CI: (1.52, 1.93) vs. PAC: HR = 2.05, 95% CI: (1.85, 2.28)). Furthermore, smoking was strongest associated with mortality-trained proteomics-based biomarkers, with ProtMort showing the highest effect size (ProtMort: β = 1.00, 95% CI: (0.88, 1.13) vs. Protein Mortality score: β = 0.92, 95% CI: (0.79, 1.05); PAC: β = 0.98, 95% CI: (0.86, 1.05)).

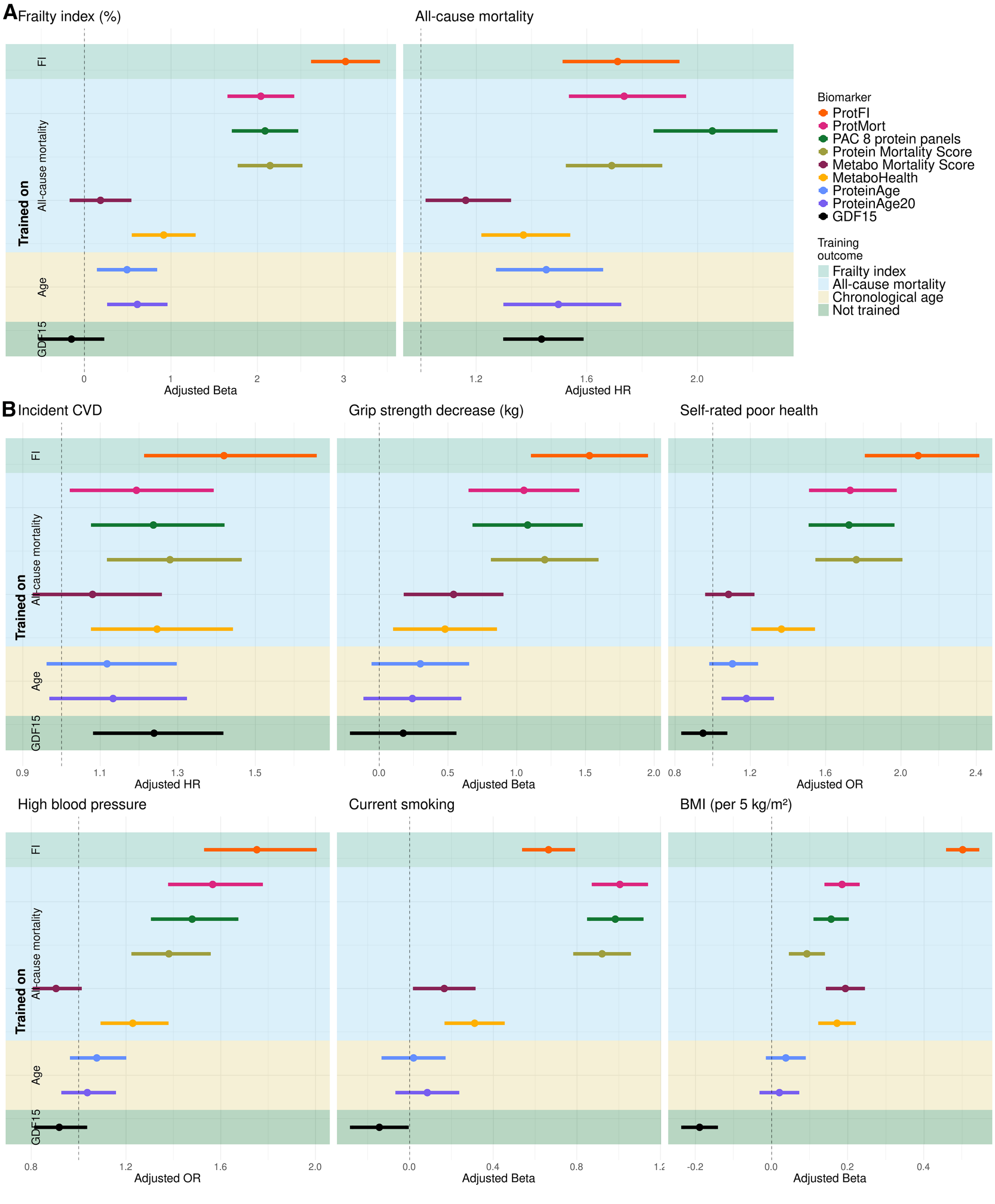

Figure 5. Comparison of associations of ProtFI and ProtMort with previously published aging biomarkers

The figure shows associations of different aging biomarkers with: (A) the training endpoints, namely the FI and all-cause mortality and (B) other age-related health indicators. Dots represent the point estimates. Error bars are displayed to show the 95% CI. The background color indicates the training outcome, with green (upper) for the FI and blue (second) for all-cause mortality, yellow (third) chronological age, and dark green (bottom) GDF15, a non-trained aging biomarker. Training outcome is also displayed on the *y* axis. Adjusted beta and OR are estimated in the independent UKB validation set. See also Table S4.

Validation in external cohorts shows generalizability of biomarkers

We validated our aging biomarkers in two independent external cohorts, the RS and the LLS. Given that the CMB panel was the only proteomic dataset available in these cohorts, our validation efforts focus on aging biomarkers trained on this subset of proteins, as well as ProtFI and ProtMort. External validation in these cohorts demonstrated that biomarkers trained using FFS-selected

proteins exhibited effect sizes comparable to those obtained using the full CMB panel (all pFDR > 0.60), as shown in Figure S3 and Table S4.

Consistent with our approach in the UKB, we also compared ProtFI and ProtMort with the metabolomics-based aging biomarker MetaboHealth. Additionally, in a subset of RS, we extended our comparisons to epigenetic biomarkers trained on chronological age[11,40] or mortality-associated phenotypes,[20] providing a broader context for evaluating their performance relative to well-established aging biomarkers.

Figure 6 (Table S4) presents the associations of the aging biomarkers in both the multi-omics subset of RS and LLS, while results from the full RS cohort can be found in Table S6. Findings in these external validation cohorts were consistent with those in the UKB, with ProtFI demonstrating stronger associations with aging-related phenotypes compared to age-trained epigenetic biomarkers, including DNAm Horvath and DNAm Hannum. In particular, ProtFI exhibited the strongest associations with aging phenotypes regardless of whether they were self-reported or objectively measured. The only exception was the association with current smoking, which was consistent with our findings in the UKB. These results highlight the generalizability of ProtFI and ProtMort to an independent cohort and across diverse populations, reinforcing their ability to robustly reflect and predict the aging process.

DISCUSSION

This study systematically compared aging biomarker development with respect to model selection, training outcome, and input data. As a result, we present ProtFI, a proteomics-based aging biomarker trained to predict the FI, showing promising associations with various health indicators.

We did not observe clear advantages of neural networks over simpler linear models. Furthermore, when training on either the FI or all-cause mortality and evaluating associations with the same outcome, biomarkers performed best when including as many proteins as possible. When assessing associations with outcomes other than the training outcome (e.g., handgrip strength or self-rated poor health), however, biomarkers trained on the FI outperformed those trained on mortality, suggesting that the FI may provide a more comprehensive representation of biological aging. Moreover, simpler biomarkers based on a subset of CMB proteins or a small number of proteins selected through FFS showed broadly similar associations with health outcomes beyond the training outcomes compared to aging biomarkers trained on the full proteomics set. In fact, when predicting outcomes different from the one the biomarkers were trained on, such as using frailty-trained biomarkers to predict mortality, those based on the full set of proteins performed slightly worse than the CMB ones, suggesting that the full proteomics set biomarkers may be somewhat overtrained or capture less relevant features. We introduced two biomarkers, ProtFI and ProtMort, which are based on a minimal set of proteins—27 for ProtFI and 20 for ProtMort—from the forward feature selected protein set to predict the FI and mortality, respectively. ProtFI outperformed established molecular aging biomarkers including methylation, metabolomic, and previously published proteomic biomarkers in association with aging phenotypes. Furthermore, the association of ProtFI with all-cause mortality outperformed those of a previously developed frailty polygenetic risk score where a SD increase resulted in 8% additional all-cause mortality risk,[34] compared to 71%, 89%, and 65% for ProtFI in the UKB, RS, and LLS, respectively. Importantly, after adjusting ProtFI for the cross-sectional FI, the association with all-cause mortality remained strong. This indicates that ProtFI captures prognostic information on the aging process beyond the conventional FI. Finally, both CMB- and FFS-based biomarkers were validated in independent cohorts, supporting the robustness and generalizability of their associations across populations.

Our findings reveal that within this dataset, the use of more complex models, such as neural networks, did not improve predictive performance for age-related clinical phenotypes in broad. This result is consistent with existing literature suggesting that neural networks struggle with tabular data, where traditional statistical models or simpler machine learning approaches tend to perform more effective.[41] While deep learning for tabular data remains an active area of research, with recent advances exploring the use of foundation models for such data,[42] further developments are needed to assess its broader applicability in this context. Furthermore, our frailty models were trained using a mean squared error loss function. While this is the standard choice for regression models, it does assume a Gaussian distribution of the residual, which in case of frailty, is not the case, since frailty is constrained between 0 and 1. Exploring adaptations of the loss function to take this constraint into account could be an interesting avenue to augment our frailty-based models.

Training biomarkers on the FI resulted in superior predictive performance for most endpoints, even for those not directly related to the FI, such as handgrip strength and incident cardiovascular disease. This is most likely due to the broader and more nuanced nature of frailty as a training target compared to mortality. We specifically chose the FI as a training outcome because it can detect differences even among relatively healthy individuals, in contrast to more physical frailty measures such as the frailty phenotype, which categorizes individuals as frail or not frail per component.[43–46] However, we did not compare predictive performance on various health outcomes among biomarkers trained on different frailty measures, which would be an important direction for future research. For outcomes with a strong association to mortality, such as smoking,[47,48] biomarkers trained directly on mortality outperformed those trained on frailty. This finding underscores the importance of determining the objective of developing an aging biomarker in advance, as the choice of training target should align with the biomarker's intended function. It is important to note that differences in statistical power between mortality and FI-based models may also influence these results, as the number of mortality cases impacts the training power of mortality, and the high degree of censoring in the training data could affect model training and evaluation.

A similar influence of power is likely reflected in our downstream analyses: BMI was consistently associated with higher ProtFI scores across all cohorts, whereas associations with incident CVD reached significance only in the UKB and has lower effect size in LLS. This likely reflects the longer follow-up and

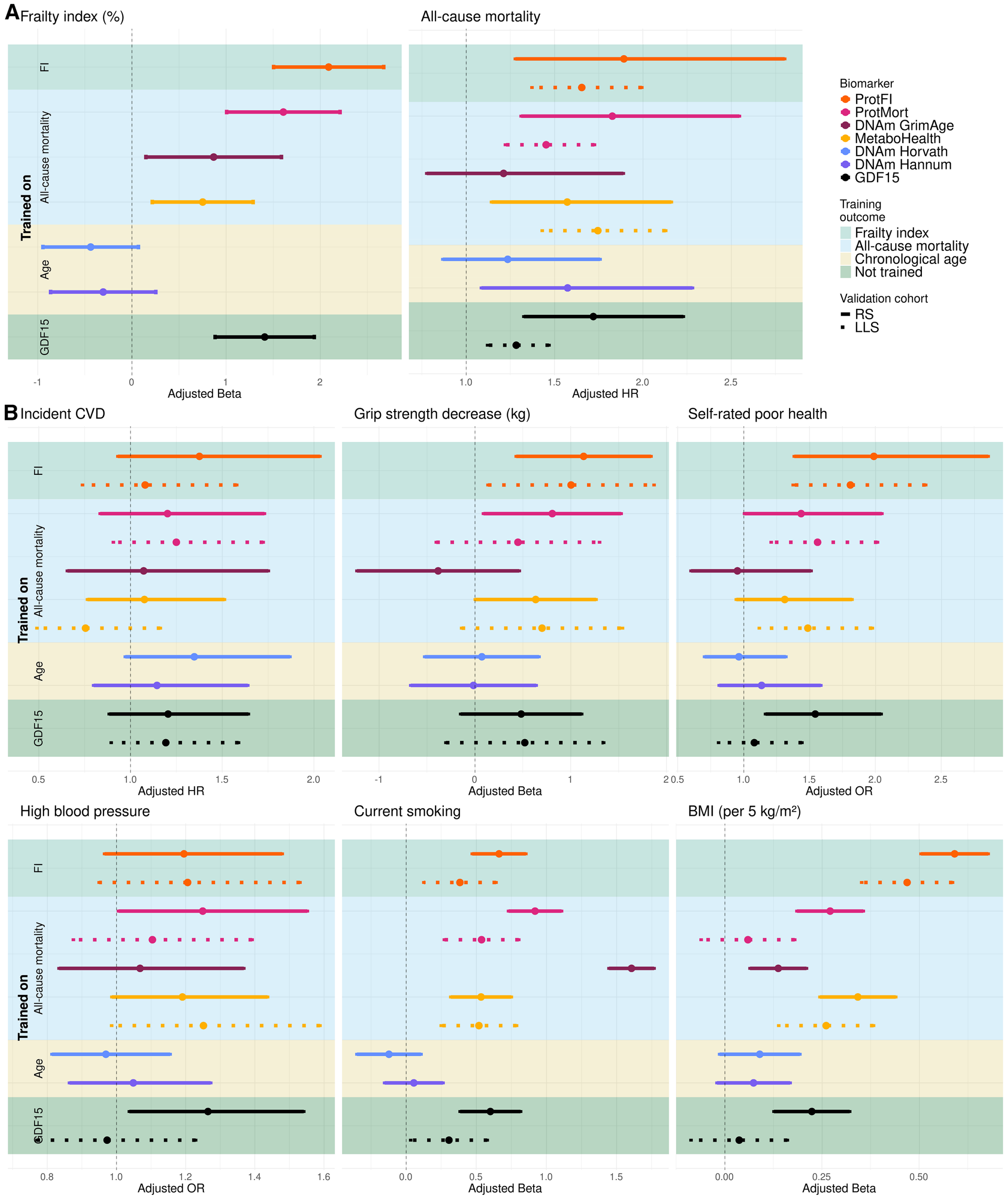

Figure 6. External validation of aging biomarkers in LLS and RS with different health outcomes

The figure shows associations of different aging biomarkers with: (A) the training endpoints, namely the FI and all-cause mortality and (B) other age-related health indicators. Dots represent the point estimates. Solid and dotted error bars indicate 95% CIs for the RS and LLS, respectively. The background color indicates the training outcome, with green (upper) for the FI and blue (second) for all-cause mortality, only in RS, yellow (third for RS) chronological age, and dark green (bottom) GDF15, a non-trained aging biomarker. Training outcome is also displayed on the *y* axis. See also Tables S4 and S6.

greater number of CVD cases in UKB, rather than a lack of relevance of ProtFI to cardiovascular health. Supporting this, ProtFI was associated with prevalent CVD in all cohorts, with the strongest effects in UKB, partly due to the inclusion of CMB conditions in the FI. In contrast, BMI is consistently measured, which may explain its robust association with ProtFI.

Besides the selection of training target, our results emphasize the significance of considering the intended use of a biomarker when choosing the number of proteins to include. In both our internal validation set as well as two independent validation cohorts, increasing the number of proteins generally improved performance for the specific endpoint the biomarker was trained on,

such as mortality or frailty. However, this improvement did not always lead to better associations with other health outcomes. In some cases, the point estimates of the FFS biomarkers were even higher than those of CMB. This may be explained by the targeted selection of proteins in FFS that are more consistently associated with age-related decline, which may have improved the signal-to-noise ratio by excluding proteins with weaker or more diffuse associations.

While associations with aging-related health outcomes remained or even increased when narrowing down the protein selection, the relevant goodness-of-fit metric (e.g., the concordance index or R^2 value) were lower. Moreover, the scoring of these metrics was rather low in general. However, when performing association analysis, our biomarkers compared competitively to existing markers. This highlights the point that a lower goodness-of-fit score (e.g., the concordance index or R^2 value) does not necessarily indicate that the biomarker performs poorly for other health outcomes. While these metrics are useful for model comparison, they should not be the sole criterion for evaluating a biomarker's relevance in risk assessment, particularly in population health management or clinical settings. Reducing the number of proteins, for example, may lower costs and facilitate broader implementation in the population, even if it slightly diminishes predictive accuracy in a population-based context. Future research should carefully balance the trade-off between accuracy and practicality, considering the biomarker's translational potential in real-world applications.

In our study, only one of the 43 proteins included in ProtFI and/or ProtMort showed a possible correspondence with previously reported frailty-related genetic loci (MET, Mak et al.[34]). This limited overlap is expected, as genetic associations reflect inherited predisposition, whereas circulating proteins capture downstream biological processes shaped by environmental exposures, comorbidities, and aging. Given the modest heritability of frailty and the restricted proteomic coverage in our replication panel (344 proteins), substantial overlap with GWAS findings is unlikely. Moreover, our feature-selection approach optimizes predictive performance rather than biological completeness, leading to the exclusion of highly correlated or redundant proteins. Thus, the low overlap with genetic loci does not diminish the relevance of the identified proteins; instead, it suggests that proteomic signatures provide complementary information for prediction of frailty and related mortality.

Although previous studies have shown differences between men and women when selecting proteins associated with a disease (e.g., in the integration of a biomarker PRS on the standard PRS for prediction of coronary heart disease[49]), we decided not to stratify our biomarkers by sex. This decision was based on our analyses that showed no differences in model performance when stratifying by sex. The proteins that were selected by sex-specific models were, however, largely non-overlapping. While this might suggest underlying differences in biological mechanisms, the causes for this difference are equally likely due to modeling limitations (that filters correlated proteins). We therefore decided to abstain from biological interpretation of the differences in protein selection between men and women, and focus on optimizing for predictive performance when deciding whether to stratify or not.

In our study, multi-omics biomarkers did not outperform single-omics biomarkers in terms of predictive accuracy. However, both MetaboHealth and protein-based biomarkers demonstrated independent associations with outcomes, suggesting that integrating multiple omics layers may still offer valuable complementary insights. This inability to create multi-omics aging biomarkers that consistently outperform single-*omic* aging biomarkers developed on one molecular layer is consistent with previous efforts to create multi-omics aging biomarkers.[38] The added noise from integrating different molecular layers may dilute the additional information provided by these layers. Furthermore, our results align with previous work emphasizing that multi-omics approaches may enhance biological understanding rather than directly improving predictive performance.[50,51] As the field of multi-omics integration methods is rapidly evolving, its potential to improve aging biomarker performance may change in the future.

Besides the cost reductions associated with using fewer proteins, selecting a minimal set of proteins may also enhance robustness. By focusing only on proteins strongly associated with the training target, we reduce the risk of overfitting to noisy or less relevant proteins, thereby improving the generalizability of the biomarker to external cohorts. Our external validation results in the RS and LLS cohorts support this hypothesis, further highlighting the benefits of using a smaller, carefully selected protein set.

In conclusion, this study presents a framework to systematically develop and compare aging biomarkers. Our approach underscores the utility of biomarkers that are designed to predict outcomes beyond those used in their training. We introduce robust aging biomarkers, ProtFI and ProtMort, which trained on only 27 and 20 proteins, demonstrate that using more proteins or more complex models does not necessarily lead to a better biomarker performance. Furthermore, our results highlight that in a rapidly expanding field, generalizability, and external validation must take priority in aging biomarker development. This study provides a foundation for future research on aging biomarkers and potential applications in both population health and clinical settings.

Limitations of the study

Several limitations of the current study warrant consideration. The UKB is relatively young and healthy, resulting in lower statistical power for mortality-based biomarkers and a less frail population for FI prediction. This raises questions about the generalizability of these results to diseased or older populations. However, the external validation in older cohorts (RS and LLS) demonstrates the robustness of the aging biomarkers developed in this study. With the exception of MetaboHealth, all aging biomarkers we compared were developed using data from the UKB. On the other hand, RS and LLS were included in the training of MetaboHealth, accounting for 13.7% of its training set.[13] The stronger associations of MetaboHealth in LLS and RS compared to UKB, and the reverse trend for the other biomarkers we compared, suggest that the effect sizes observed for the other biomarkers may be inflated due to overlap with their training data. Additionally, while we incorporate Olink proteomic and ^{1}H-NMR metabolomic information, we did not include other

CellPress
OPEN ACCESS

molecular layers to fully assess the robustness of our methodological findings across different molecular platforms. Finally, although our predictors show robust associations with frailty, mortality, and age-related disease, these outcomes reflect age-related health status rather than the underlying biological aging process. Our study therefore does not make claims about the biological mechanisms of aging. Instead, the models capture variation in age-related health risk within the available cohorts. Moreover, our findings regarding model performance are specific to these datasets; different molecular platforms and cohort characteristics may favor different modeling approaches. We, therefore, recommend that future aging biomarker development should systematically compare a broad range of models rather than relying on a single preferred approach and compare these models against existing aging biomarkers.

RESOURCE AVAILABILITY

Lead contact

Requests for further information and resources should be directed to and will be fulfilled by the lead contact, Joyce van Meurs (j.vanmeurs@erasmusmc.nl).

Materials availability

This study did not generate new unique reagents.

Data and code availability

- Data from the UK Biobank can be accessed through a procedure described at https://www.ukbiobank.ac.uk/enable-your-research.
- The data from the Leiden Longevity Study and the Rotterdam Study are accessible for researchers upon request. Please find the required forms for the Leiden Longevity Study at: https://leidenlangleven.nl/data-access/. Requests for Rotterdam Study data should be directed toward the management team of the Rotterdam Study (datamanagement.ergo@erasmusmc.nl), which has a protocol for approving data requests. Because of restrictions based on privacy regulations and informed consent of the participants, data cannot be made freely available in a public repository.
- Python and R code for reproducing the results in this manuscript as well as an R package to compute ProtFI and ProtMort can be found at https://github.com/swiergarst/ProtFI and https://doi.org/10.5281/zenodo.18982933.
- Any additional information required to reanalyze the data reported in this paper is available from the lead contact upon request.

ACKNOWLEDGMENTS

The authors want to thank M. Austin Argentieri for the extensive help with the ProteinAge biomarker. This research has been conducted using the UK Biobank Resource under application number 67864. The Rotterdam Study is funded by the Erasmus Medical Center and Erasmus University, Rotterdam; the Netherlands Organization for the Health Research and Development (ZonMw); the Research Institute for Diseases in the Elderly (RIDE); the Ministry of Education, Culture and Science; the Ministry for Health, Welfare and Sports, the European Commission (DG XII); and the Municipality of Rotterdam. The authors are grateful to the study participants, the staff from the Rotterdam Study and the participating general practitioners and pharmacists. The Leiden Longevity Study is supported by the European Union's Seventh Framework Programme (FP7/2007–2011) under grant agreement number 259679. This study was financially supported by the Innovation-Oriented Research Program on Genomics (SenterNovem IGE05007), the Centre for Medical Systems Biology, and the Netherlands Consortium for Healthy Ageing (grant 050-060-810), all in the framework of the Netherlands Genomics Initiative, Netherlands Organisation for Scientific Research, by BBMRI-NL, a Research Infrastructure financed by the Dutch government (NWO 184.021.007 and 184.033.111). This research was supported by the Netherlands Organisation for Health Research and Development (research grant 457001001 for the VOILA project).

AUTHOR CONTRIBUTIONS

Conceptualization, S.G., L.K., M.R., E.S., and J.v.M.; methodology, S.G., L.K., E.v.d.A., M.B., M.R., E.S., and J.v.M.; software, validation, formal analyses, writing – original draft, visualization, S.G. and L.K.; data curation, N.v.d.B., M.G., S.M., M.B., E.S., and J.v.M.; writing – review and editing, E.v.d.A., N.v.d.B., M.G., S.M., M.B., M.R., E.S., and J.v.M.; supervision, M.R., E.S., and J.v.M.; funding acquisition, E.v.d.A., S.M., M.B., M.R., E.S., and J.v.M.

DECLARATION OF INTERESTS

The authors declare no competing interests.

STAR★METHODS

Detailed methods are provided in the online version of this paper and include the following:

- KEY RESOURCES TABLE
- EXPERIMENTAL MODEL AND STUDY PARTICIPANT DETAILS
  - UK Biobank
  - Rotterdam Study
  - Leiden Longevity Study
- METHOD DETAILS
  - Data preprocessing
  - Ascertainment of covariates and outcomes
  - Biomarker models
  - Multi-omics
  - Forward feature selection
  - Calculation of previously developed aging biomarkers
  - Association analysis
- QUANTIFICATION AND STATISTICAL ANALYSIS
  - Statistical model comparison

SUPPLEMENTAL INFORMATION

Supplemental information can be found online at https://doi.org/10.1016/j.crmeth.2026.101405.

Received: October 16, 2025
Revised: January 22, 2026
Accepted: March 18, 2026
Published: April 10, 2026

REFERENCES

1. Partridge, L., Deelen, J., and Slagboom, P.E. (2018). Facing up to the global challenges of ageing. Nature *561*, 45–56. https://doi.org/10.1038/s41586-018-0457-8.
2. Niccoli, T., and Partridge, L. (2012). Ageing as a risk factor for disease. Curr. Biol. *22*, R741–R752. https://doi.org/10.1016/j.cub.2012.07.024.
3. Lowsky, D.J., Olshansky, S.J., Bhattacharya, J., and Goldman, D.P. (2014). Heterogeneity in Healthy Aging. J. Gerontol. A Biol. Sci. Med. Sci. *69*, 640–649. https://doi.org/10.1093/gerona/glt162.
4. Rutledge, J., Oh, H., and Wyss-Coray, T. (2022). Measuring biological age using omics data. Nat. Rev. Genet. *23*, 715–727. https://doi.org/10.1038/s41576-022-00511-7.
5. Félix, J., Martínez de Toda, I., Díaz-Del Cerro, E., González-Sánchez, M., and De la Fuente, M. (2024). Frailty and biological age. Which best describes our aging and longevity? Mol. Aspects Med. *98*, 101291. https://doi.org/10.1016/j.mam.2024.101291.

6. Clegg, A., Young, J., Iliffe, S., Rikkert, M.O., and Rockwood, K. (2013). Frailty in elderly people. Lancet *381*, 752–762. https://doi.org/10.1016/S0140-6736(12)62167-9.
7. Dent, E., Kowal, P., and Hoogendijk, E.O. (2016). Frailty measurement in research and clinical practice: A review. Eur. J. Intern. Med. *31*, 3–10. https://doi.org/10.1016/j.ejim.2016.03.007.
8. Moqri, M., Herzog, C., Poganik, J.R., , et al.; Biomarkers of Aging Consortium; Justice, J., Belsky, D.W., Higgins-Chen, A., Moskalev, A., Fuellen, G., Cohen, A.A. (2023). Biomarkers of aging for the identification and evaluation of longevity interventions. Cell *186*, 3758–3775. https://doi.org/10.1016/j.cell.2023.08.003.
9. Williams, D.M., Jylhävä, J., Pedersen, N.L., and Hägg, S. (2019). A Frailty Index for UK Biobank Participants. J. Gerontol.: Series A *74*, 582–587. https://doi.org/10.1093/gerona/gly094.
10. Mitnitski, A.B., Mogilner, A.J., and Rockwood, K. (2001). Accumulation of deficits as a proxy measure of aging. Sci. World J. *1*, 323–336. https://doi.org/10.1100/tsw.2001.58.
11. Horvath, S. (2013). DNA methylation age of human tissues and cell types. Genome Biol. *14*, R115. https://doi.org/10.1186/gb-2013-14-10-r115.
12. Horvath, S., and Raj, K. (2018). DNA methylation-based biomarkers and the epigenetic clock theory of ageing. Nat. Rev. Genet. *19*, 371–384. https://doi.org/10.1038/s41576-018-0004-3.
13. Deelen, J., Kettunen, J., Fischer, K., van der Spek, A., Trompet, S., Kastenmüller, G., Boyd, A., Zierer, J., van den Akker, E.B., Ala-Korpela, M., et al. (2019). A metabolic profile of all-cause mortality risk identified in an observational study of 44,168 individuals. Nat. Commun. *10*, 3346. https://doi.org/10.1038/s41467-019-11311-9.
14. Peters, M.J., Joehanes, R., Pilling, L.C., Schurmann, C., Conneely, K.N., Powell, J., Reinmaa, E., Sutphin, G.L., Zhernakova, A., Schramm, K., et al. (2015). The transcriptional landscape of age in human peripheral blood. Nat. Commun. *6*, 8570. https://doi.org/10.1038/ncomms9570.
15. Tanaka, T., Basisty, N., Fantoni, G., Candia, J., Moore, A.Z., Biancotto, A., Schilling, B., Bandinelli, S., and Ferrucci, L. (2020). Plasma proteomic biomarker signature of age predicts health and life span. eLife *9*, e61073. https://doi.org/10.7554/eLife.61073.
16. Galkin, F., Mamoshina, P., Aliper, A., Lane, E., Moskalev, V., Gladyshev, V.N., and Zhavoronkov, A. (2020). Human Gut Microbiome Aging Clock Based on Taxonomic Profiling and Deep Learning. iScience *23*, 101199. https://doi.org/10.1016/j.isci.2020.101199.
17. Würtz, P., Kangas, A.J., Soininen, P., Lawlor, D.A., Davey Smith, G., and Ala-Korpela, M. (2017). Quantitative Serum Nuclear Magnetic Resonance Metabolomics in Large-Scale Epidemiology: A Primer on -Omic Technologies. Am. J. Epidemiol. *186*, 1084–1096. https://doi.org/10.1093/aje/kwx016.
18. Lehallier, B., Gate, D., Schaum, N., Nanasi, T., Lee, S.E., Yousef, H., Moran Losada, P., Berdnik, D., Keller, A., Verghese, J., et al. (2019). Undulating changes in human plasma proteome profiles across the lifespan. Nat. Med. *25*, 1843–1850. https://doi.org/10.1038/s41591-019-0673-2.
19. Eiriksdottir, T., Ardal, S., Jonsson, B.A., Lund, S.H., Ivarsdottir, E.V., Norland, K., Ferkingstad, E., Stefansson, H., Jonsdottir, I., Holm, H., et al. (2021). Predicting the probability of death using proteomics. Commun. Biol. *4*, 758. https://doi.org/10.1038/s42003-021-02289-6.
20. Lu, A.T., Quach, A., Wilson, J.G., Reiner, A.P., Aviv, A., Raj, K., Hou, L., Baccarelli, A.A., Li, Y., Stewart, J.D., et al. (2019). DNA methylation GrimAge strongly predicts lifespan and healthspan. Aging (Albany NY) *11*, 303–327. https://doi.org/10.18632/aging.101684.
21. McCrory, C., Fiorito, G., Hernandez, B., Polidoro, S., O'Halloran, A.M., Hever, A., Ni Cheallaigh, C., Lu, A.T., Horvath, S., Vineis, P., and Kenny, R.A. (2021). GrimAge Outperforms Other Epigenetic Clocks in the Prediction of Age-Related Clinical Phenotypes and All-Cause Mortality. J. Gerontol. A Biol. Sci. Med. Sci. *76*, 741–749. https://doi.org/10.1093/gerona/glaa286.
22. Kuiper, L.M., Polinder-Bos, H.A., Bizzarri, D., Vojinovic, D., Vallerga, C.L., Beekman, M., Dollé, E.T., Ghanbari, M., Voortman, T., Reinders, M.J.T., et al. (2023). Epigenetic and Metabolomic Biomarkers for Biological Age: A Comparative Analysis of Mortality and Frailty Risk. J. Gerontol. A Biol. Sci. Med. Sci. *78*, 1753–1762. https://doi.org/10.1093/gerona/glad137.
23. Leme, D.E.d.C., and De Oliveira, C. (2023). Machine Learning Models to Predict Future Frailty in Community-Dwelling Middle-Aged and Older Adults: The ELSA Cohort Study. J. Gerontol. A Biol. Sci. Med. Sci. *78*, 2176–2184. https://doi.org/10.1093/gerona/glad127.
24. Yan, L., Ren, E., Guo, C., Peng, Y., Chen, H., and Li, W. (2025). Development and validation of a predictive model for frailty risk in older adults with cardiovascular-metabolic comorbidities. Front. Public Health *13*, 1561845. https://doi.org/10.3389/fpubh.2025.1561845.
25. Wu, Y., Jia, M., Xiang, C., and Fang, Y. (2022). Latent trajectories of frailty and risk prediction models among geriatric community dwellers: an interpretable machine learning perspective. BMC Geriatr. *22*, 900. https://doi.org/10.1186/s12877-022-03576-5.
26. Sathyan, S., Ayers, E., Gao, T., Milman, S., Barzilai, N., and Verghese, J. (2020). Plasma proteomic profile of frailty. Aging Cell *19*, e13193. https://doi.org/10.1111/acel.13193.
27. Jia, X., Gao, W., Zhang, J., Zhao, Y., Zhang, L., Cao, X., Wu, Y., Ma, L., Chen, L., Sun, L., et al. (2026). Plasma Proteomic Signature of Frailty in 50,506 Adults. Cell Metabolism. https://doi.org/10.1016/j.cmet.2026.02.013.
28. Sudlow, C., Gallacher, J., Allen, N., Beral, V., Burton, P., Danesh, J., Downey, P., Elliott, P., Green, J., Landray, M., et al. (2015). UK Biobank: An Open Access Resource for Identifying the Causes of a Wide Range of Complex Diseases of Middle and Old Age. PLoS Med. *12*, e1001779. https://doi.org/10.1371/journal.pmed.1001779.
29. Sun, B.B., Chiou, J., Traylor, M., Benner, C., Hsu, Y.H., Richardson, T.G., Surendran, P., Mahajan, A., Robins, C., Vasquez-Grinnell, S.G., et al. (2023). Plasma proteomic associations with genetics and health in the UK Biobank. Nature *622*, 329–338. https://doi.org/10.1038/s41586-023-06592-6.
30. Ikram, M.A., Kieboom, B.C.T., Brouwer, W.P., Brusselle, G., Chaker, L., Ghanbari, M., Goedegebure, A., Ikram, M.K., Kavousi, M., de Knegt, R.J., et al. (2024). The Rotterdam Study. Design update and major findings between 2020 and 2024. Eur. J. Epidemiol. *39*, 183–206. https://doi.org/10.1007/s10654-023-01094-1.
31. Schoenmaker, M., de Craen, A.J.M., de Meijer, P.H.E.M., Beekman, M., Blauw, G.J., Slagboom, P.E., and Westendorp, R.G.J. (2006). Evidence of genetic enrichment for exceptional survival using a family approach: The Leiden Longevity Study. Eur. J. Hum. Genet. *14*, 79–84. https://doi.org/10.1038/sj.ejhg.5201508.
32. Fry, A., Littlejohns, T.J., Sudlow, C., Doherty, N., Adamska, L., Sprosen, T., Collins, R., and Allen, N.E. (2017). Comparison of Sociodemographic and Health-Related Characteristics of UK Biobank Participants With Those of the General Population. Am. J. Epidemiol. *186*, 1026–1034. https://doi.org/10.1093/aje/kwx246.
33. Atkins, J.L., Jylhävä, J., Pedersen, N.L., Magnusson, P.K., Lu, Y., Wang, Y., Hägg, S., Melzer, D., Williams, D.M., and Pilling, L.C. (2021). A genome-wide association study of the frailty index highlights brain pathways in ageing. Aging Cell *20*, e13459. https://doi.org/10.1111/acel.13459.
34. Mak, J.K.L., Qin, C., Krüger, M., Kuukka, A., Lehto, P., Southerington, T., Smith, S., Tammerluoto, J., Vähätalo, I., Luukkaala, T., et al. (2025). Large-scale genome-wide analyses with proteomics integration reveal novel loci and biological insights into frailty. Nat. Aging *5*, 1589–1600. https://doi.org/10.1038/s43587-025-00925-y.
35. Rosoff, D.B., Mavromatis, L.A., Bell, A.S., Wagner, J., Jung, J., Marioni, R.E., Davey Smith, G., Horvath, S., and Lohoff, F.W. (2023). Multivariate genome-wide analysis of aging-related traits identifies novel loci and new drug targets for healthy aging. Nat. Aging *3*, 1020–1035. https://doi.org/10.1038/s43587-023-00455-5.
36. Ye, Y., Noche, R.B., Szejko, N., Both, C.P., Acosta, J.N., Leasure, A.C., Brown, S.C., Sheth, K.N., Gill, T.M., Zhao, H., and Falcone, G.J. (2023).

A genome-wide association study of frailty identifies significant genetic correlation with neuropsychiatric, cardiovascular, and inflammation pathways. GeroScience *45*, 2511–2523. https://doi.org/10.1007/s11357-023-00771-z.

37. Argentieri, M.A., Xiao, S., Bennett, D., Winchester, L., Nevado-Holgado, A.J., Ghose, U., Albukhari, A., Yao, P., Mazidi, M., Lv, J., et al. (2024). Proteomic aging clock predicts mortality and risk of common age-related diseases in diverse populations. Nat. Med. *30*, 2450–2460. https://doi.org/10.1038/s41591-024-03164-7.
38. Gadd, D.A., Hillary, R.F., Kuncheva, Z., Mangelis, T., Cheng, Y., Dissanayake, M., Admanit, R., Gagnon, J., Lin, T., Ferber, K.L., et al. (2024). Blood protein assessment of leading incident diseases and mortality in the UK Biobank. Nat. Aging *4*, 939–948. https://doi.org/10.1038/s43587-024-00655-7.
39. Kuo, C.-L., Chen, Z., Liu, P., Pilling, L.C., Atkins, J.L., Fortinsky, R.H., Kuchel, G.A., and Diniz, B.S. (2024). Proteomic aging clock (PAC) predicts age-related outcomes in middle-aged and older adults. Aging Cell *23*, e14195. https://doi.org/10.1111/acel.14195.
40. Hannum, G., Guinney, J., Zhao, L., Zhang, L., Hughes, G., Sadda, S., Klotzle, B., Bibikova, M., Fan, J.-B., Gao, Y., et al. (2013). Genome-wide Methylation Profiles Reveal Quantitative Views of Human Aging Rates. Mol. Cell *49*, 359–367. https://doi.org/10.1016/j.molcel.2012.10.016. Genome-wide.
41. Borisov, V., Leemann, T., Sebler, K., Haug, J., Pawelczyk, M., and Kasneci, G. (2024). Deep Neural Networks and Tabular Data: A Survey. IEEE Trans. Neural Netw. Learn. Syst. *35*, 7499–7519. https://doi.org/10.1109/TNNLS.2022.3229161.
42. Hollmann, N., Müller, S., Purucker, L., Krishnakumar, A., Körfer, M., Hoo, S.B., Schirrmeister, R.T., and Hutter, F. (2025). Accurate predictions on small data with a tabular foundation model. Nature *637*, 319–326. https://doi.org/10.1038/s41586-024-08328-6.
43. Fried, L.P., Tangen, C.M., Walston, J., Newman, A.B., Hirsch, C., Gottdiener, J., Seeman, T., Tracy, R., Kop, W.J., Burke, G., et al. (2001). Frailty in Older Adults: Evidence for a Phenotype. J. Gerontol. A Biol. Sci. Med. Sci. *56*, 146–156. https://doi.org/10.1093/gerona/56.3.M146.
44. Theou, O., Brothers, T.D., Mitnitski, A., and Rockwood, K. (2013). Operationalization of frailty using eight commonly used scales and comparison of their ability to predict all-cause mortality. J. Am. Geriatr. Soc. *61*, 1537–1551. https://doi.org/10.1111/jgs.12420.
45. Rockwood, K., and Mitnitski, A. (2007). Frailty in relation to the accumulation of deficits. J. Gerontol. A Biol. Sci. Med. Sci. *62*, 722–727. https://doi.org/10.1093/gerona/62.7.722.
46. Blodgett, J., Theou, O., Kirkland, S., Andreou, P., and Rockwood, K. (2015). Frailty in NHANES: Comparing the frailty index and phenotype. Arch. Gerontol. Geriatr. *60*, 464–470. https://doi.org/10.1016/j.archger.2015.01.016.
47. Ezzati, M., and Lopez, A.D. (2003). Estimates of global mortality attributable to smoking in 2000. Lancet *362*, 847–852. https://doi.org/10.1016/S0140-6736(03)14338-3.
48. Reitsma, M.B., Flor, L.S., Mullany, E.C., Gupta, V., Hay, S.I., and Gakidou, E. (2021). Spatial, temporal, and demographic patterns in prevalence of smoking tobacco use and initiation among young people in 204 countries and territories, 1990-2019. Lancet Public Health *6*, e472–e481. https://doi.org/10.1016/S2468-2667(21)00102-X.
49. Lin, J., Mars, N., Fu, Y., Ripatti, P., Kiiskinen, T., FinnGen study; Tukiainen, T., Ripatti, S., and Pirinen, M. (2023). Integration of Biomarker Polygenic Risk Score Improves Prediction of Coronary Heart Disease. JACC, Basic Transl. Sci. *8*, 1489–1499. https://doi.org/10.1016/j.jacbts.2023.07.006.
50. Li, Y., Herold, T., Mansmann, U., and Hornung, R. (2024). Does combining numerous data types in multi-omics data improve or hinder performance in survival prediction? Insights from a large-scale benchmark study. BMC Med. Inform. Decis. Mak. *24*, 244. https://doi.org/10.1186/s12911-024-02642-9.
51. Bizzarri, D., Reinders, M.J.T., Kuiper, L., Beekman, M., Deelen, J., van Meurs, J.B.J., van Dongen, J., Pool, R., Boomsma, D.I., Ghanbari, M., et al. (2024). NMR metabolomics-guided DNA methylation mortality predictors. EBioMedicine *107*, 105279. https://doi.org/10.1016/j.ebiom.2024.105279.
52. Bizzarri, D., Reinders, M.J.T., Beekman, M., Slagboom, P.E., and van den Akker, E.B.; BbmriNl (2023). Technical report: A comprehensive comparison between different quantification versions of Nightingale Health's 1H-NMR metabolomics platform. Metabolites *13*, 1181. https://doi.org/10.3390/metabo13121181.
53. Schoufour, J.D., Erler, N.S., Jaspers, L., Kiefte-de Jong, J.C., Voortman, T., Ziere, G., Lindemans, J., Klaver, C.C., Tiemeier, H., Stricker, B., et al. (2017). Design of a frailty index among community living middle-aged and older people: The Rotterdam study. Maturitas *97*, 14–20. https://doi.org/10.1016/j.maturitas.2016.12.002.
54. Rietveld, C.A., Medland, S.E., Derringer, J., Yang, J., Esko, T., Martin, N.W., Westra, H.-J., Shakhbazov, K., Abdellaoui, A., Agrawal, A., et al. (2013). GWAS of 126,559 Individuals Identifies Genetic Variants Associated with Educational Attainment. Science *340*, 1467–1471. https://doi.org/10.1126/science.1235488.
55. Leening, M.J.G., Kavousi, M., Heeringa, J., Van Rooij, F.J.A., Verkroost-Van Heemst, J., Deckers, J.W., Mattace-Raso, F.U.S., Ziere, G., Hofman, A., Stricker, B.H.C., and Witteman, J.C.M. (2012). Methods of data collection and definitions of cardiac outcomes in the Rotterdam Study. Eur. J. Epidemiol. *27*, 173–185. https://doi.org/10.1007/s10654-012-9668-8.
56. Friedman, J., Hastie, T., and Tibshirani, R. (2010). Regularization Paths for Generalized Linear Models via Coordinate Descent. J. Stat. Softw. *33*, 1–22. https://doi.org/10.18637/jss.v033.i01.
57. Katzman, J.L., Shaham, U., Cloninger, A., Bates, J., Jiang, T., and Kluger, Y. (2018). DeepSurv: personalized treatment recommender system using a Cox proportional hazards deep neural network. BMC Med. Res. Methodol. *18*, 24. https://doi.org/10.1186/s12874-018-0482-1.
58. Lock, E.F., Hoadley, K.A., Marron, J.S., and Nobel, A.B. (2013). Joint and individual variation explained (JIVE) for integrated analysis of multiple data types. Ann. Appl. Stat. *7*, 523–542. https://doi.org/10.1214/12-AOAS597.
59. Feng, Q., Jiang, M., Hannig, J., and Marron, J.S. (2018). Angle-based joint and individual variation explained. J. Multivar. Anal. *166*, 241–265. https://doi.org/10.1016/j.jmva.2018.03.008.
60. Bizzarri, D., Reinders, M.J.T., Beekman, M., Slagboom, P.E., and Van Den Akker, E.B. (2022). MiMIR: R-shiny application to infer risk factors and endpoints from Nightingale Health's 1H-NMR metabolomics data. Bioinformatics *38*, 3847–3849. https://doi.org/10.1093/bioinformatics/btac388.
61. Pelegí-Sisó, D., de Prado, P., Ronkainen, J., Bustamante, M., and González, J.R. (2021). methylclock: a Bioconductor package to estimate DNA methylation age. Bioinformatics *37*, 1759–1760. https://doi.org/10.1093/bioinformatics/btaa825.
62. Teschendorff, A.E., and Horvath, S. (2025). Epigenetic ageing clocks: statistical methods and emerging computational challenges. Nat. Rev. Genet. *26*, 350–368. https://doi.org/10.1038/s41576-024-00807-w.
63. Benjamini, Y., and Hochberg, Y. (1995). Controlling the False Discovery Rate: A Practical and Powerful Approach to Multiple Testing. J. Roy. Stat. Soc. B *57*, 289–300. https://doi.org/10.1111/j.2517-6161.1995.tb02031.x.

STAR★METHODS

KEY RESOURCES TABLE

REAGENT or RESOURCE	SOURCE	IDENTIFIER
**Biological samples**		
UK Biobank proteomics and metabolomics data	UK Biobank Coordinating Center	Application number 67864; https://www.ukbiobank.ac.uk
Rotterdam Study proteomics, metabolomics, and DNA methylation data	Rotterdam Study Coordinating Center	https://www.ergo-onderzoek.nl
Leiden Longevity Study proteomics and metabolomics data	Leiden Longevity Study Coordinating Center	https://www.leidenlangleven.nl
**Critical commercial assays**		
Olink® Explore 384 Cardiometabolic panel	Olink Proteomics AB	https://www.olink.com
Olink® Explore 3072	Olink Proteomics AB	https://www.olink.com
Nightingale ^{1}H-NMR Metabolomics platform	Nightingale Health Ltd	2020 and re-quantified 2020 version; https://nightingalehealth.com
**Software and algorithms**		
Python	Python Software Foundation	v3.12.1; https://www.python.org
R	R Foundation for Statistical Computing	V4.4.2; https://www.r-project.org
JupyterLab	Project Jupyter	v4.0.11; https://jupyter.org/
pandas	PyPI	v2.2.0; https://pandas.pydata.org/
NumPy	PyPI	v1.26.3; https://numpy.org/
scikit-learn	PyPI	v1.3.2; https://scikit-learn.org/
scikit-survival	PyPI	v0.22.2;https://scikit-survival.readthedocs.io/
lifelines	PyPI	v0.29.0; https://lifelines.readthedocs.io/
statsmodels	PyPI	v0.14.1; https://www.statsmodels.org
SciPy	PyPI	v0.12.1; https://scipy.org
seaborn	PyPI	v0.13.2; https://seaborn.pydata.org
PyTorch	PyTorch Foundation	v2.2.1; https://pytorch.org
Weights & Biases (wandb)	Weights & Biases Inc.	v0.16.6; https://wandb.ai
tqdm	PyPI	v.4.66.1; https://pypi.org/project/tqdm
argparse	Python Standard Library	Included in Python v3.12.1
os	Python Standard Library	Included in Python v3.12.1
re	Python Standard Library	Included in Python v3.12.1
math	Python Standard Library	Included in Python v3.12.1
random	Python Standard Library	Included in Python v3.12.1
copy (deepcopy)	Python Standard Library	Included in Python v3.12.1
dplyr	CRAN	v1.1.4; https://cran.r-project.org/package=dplyr
data.table	CRAN	v1.17.8; https://cran.r-project.org/package=data.table
tidyselect	CRAN	v1.2.1; https://cran.r-project.org/package=tidyselect
mice	CRAN	v3.19.0; https://cran.r-project.org/package=mice
stringr	CRAN	v1.6.0; https://cran.r-project.org/package=stringr
ggplot2	CRAN	v4.0.1; https://cran.r-project.org/package=ggplot2

(*Continued on next page*)

CellPress
OPEN ACCESS

Continued

REAGENT or RESOURCE	SOURCE	IDENTIFIER
ggpubr	CRAN	v0.6.2; https://cran.r-project.org/package=ggpubr
patchwork	CRAN	v1.3.2; https://cran.r-project.org/package=patchwork
readr	CRAN	v2.1.6; https://cran.r-project.org/package=readr
tidyr	CRAN	v1.3.1; https://cran.r-project.org/package=tidyr
All code including ProtBiom (custom R package)	This study	https://github.com/swiergarst/ProtFI/tree/master/ProtBiom https://doi.org/10.5281/zenodo.18982933

EXPERIMENTAL MODEL AND STUDY PARTICIPANT DETAILS

UK Biobank

The UK Biobank is a prospective cohort study consisting of 500,000 participants recruited in the United Kingdom between 2006 and 2010. Invitations were sent to approximately 9.2 million individuals aged 40–69 years who lived within 25 miles (40 km) of one of 22 assessment centers across England, Wales, and Scotland, with 5.5% taking part in the baseline assessment.[28] We limited our UKB sample to participants with Olink Explore data available at baseline and who were randomly selected from the broader UKB population (*n* = 40,696). The data in the current study were accessed under application number 67864.

The UK Biobank received approval from the National Information Governance Board for Health and Social Care and the National Health Service North West Multicentre Research Ethics Committee.

Rotterdam Study

The Rotterdam Study (RS) is a prospective population-based cohort based in Ommoord, a suburb of Rotterdam, the Netherlands. The RS began in 1990 with 7,983 participants aged 55 and older and later expanded with additional cohorts: RS-II (in 2000, 3,011 participants aged 55≤), RS-III (in 2006, 3.932 participants aged 45≤), and RS-IV (in 2016, 3,005 participants aged 40≤). Participants underwent home interviews and standardized health assessments at baseline and follow-ups every 3 to 5 years, conducted by trained research staff in a dedicated research facility.[30] For this study, we measured the Olink Explore Cardiometabolic panel in RS-III participants who attended the research facility for the second visit, prioritizing those with the greatest overlap with metabolomics, gut microbiome, and DNA methylation data measured (*n* = 996), for which participants had previously been randomly selected. One participant failed Olink quality control and was therefore excluded, leaving 995 participants. Despite the multi-omic prioritization, not all selected participants had complete data across all molecular platforms.

The Rotterdam Study has been approved by the Medical Ethics Committee of the Erasmus MC (registration number MEC 02.1015) and by the Dutch Ministry of Health, Welfare and Sport (Population Screening Act WBO, license number 1071272-159521-PG). The Rotterdam Study Personal Registration Data collection is filed with the Erasmus MC Data Protection Officer under registration number EMC1712001. All participants provided written informed consent to participate in the study and to have their information obtained from their treating physicians.

Leiden Longevity Study

The Leiden Longevity Study (LLS), conducted between 2002 and 2006, includes 420 Dutch Caucasian families with at least two long-lived full siblings (≥89 years for men, ≥91 years for women) who were willing to participate. The study enrolled 991 siblings and cousins (first generation), as well as 1,671 of their children and 744 of their children's partners (second generation).[31] For this study, we included a subset of 500 second-generation participants and their partners who attended the third measurement of whom ^{1}H-NMR-metabolomics was previously measured, and in whom the Olink Explore Cardiometabolic panel was assessed.

The Leiden Longevity Study protocol was approved by the Medical Ethical Committee of the Leiden University Medical Center before the start of the study (P09.140) at August 5th, 2009. The first participant was enrolled at August 24th, 2009. In accordance with the Declaration of Helsinki, the Leiden Longevity Study obtained informed consent from all participants prior to their entering the study.

METHOD DETAILS

Data preprocessing

Proteomic profiling

The Olink Explore assay (Olink, Uppsala, Sweden), based on the proximity extension assay technique, was used to quantify plasma levels of 2,923 proteins in the UK Biobank and 384 proteins in the Rotterdam Study and Leiden Longevity Study. The Olink Explore

3072 platform integrates eight Olink panels—Cardiometabolic, Cardiometabolic II, Inflammation, Inflammation II, Neurology, Neurology II, Oncology, and Oncology II. Based on data availability at the time of analysis and our overarching aim to maximize biomarker generalizability, we used the non-II panels for biomarker development (total of 1463 proteins before quality control). The II-panels were only included in the calculation of previously published aging biomarkers that incorporated proteins from these panels.[37,39] In the Rotterdam Study and Leiden Longevity Study, only the Cardiometabolic panel was measured. Previous proteomic studies in the UK Biobank have described the profiling conducted in that cohort.[29]

In the Rotterdam Study and Leiden Longevity Study, proteomic analysis on EDTA plasma was performed by the Genomics Core Facility (www.genomicserasmusmc.nl) in Rotterdam following the manufacturer's protocol. Data are presented as NPX (Normalized Protein eXpression) values, Olink's relative protein quantification unit on a log2 scale. NPX values are calculated from matched counts using Next-Generation Sequencing (NGS) as the readout, providing a standardized measure of protein abundance. To enhance the comparability of NPX values across cohorts, all protein measurements were Z-transformed.

Quality control

Quality control for the UKB proteomics data was done by UKB.[29] For the Rotterdam Study and Leiden Longevity Study proteomics data, the quality control followed the standard manufacturer's procedure. In short, three internal controls are added to each sample, the Incubation control, the Extension Control, and the Amplification control. The Extension Control is used for the generation of the NPX values. The Incubation Control and the Amplification Control are used to monitor the quality of assay performance, as well as the quality of individual samples. Three external controls are included in each run, Plate Control (healthy pooled plasma), Sample Control (healthy pooled plasma), and Negative Control. The Plate Control is used for data normalization, the Sample Control is used to assess potential variation between runs and plates, and the Negative Control is used to calculate the Limit of Detection for each assay and to assess potential contamination of assays. To pass the quality control, a threshold of an average of 500 reads per combination of sample and protein was used. Additionally, for each protein per plate, the deviation of the median of the Negative Controls must be ≤ 5 standard deviations. One RS-participant failed quality control and was excluded from all analyses.

In the UK Biobank, there were after quality control by the cohort 52,704 participants with Olink protein information available. We excluded participants with >2% missingness on their proteomic information, and participants with proteomic information from multiple visits. This resulted in 40,696 participants whose proteomics data was used. Subsequently, we excluded proteins with >2% missing values, resulting in the removal of 36 proteins, leaving 1428 proteins for analysis. Further missing proteins were imputed using K-nearest-neighbor imputation, using the KNNImputer function of the SKLearn package, with k = 5.

The same procedure was followed in both external cohorts. In both RS and LLS, no participants were excluded due to missingness. However, three proteins that were not included in either ProtFI or ProtMort, namely BMP6, EPHX2, and PGLYRP1, failed the quality control in both studies. These proteins have been imputed by the proteins with the highest correlations to these proteins in the UK Biobank, respectively PDGFA, GSTA1, and RETN (see Table S7 for a full list of highest correlating proteins in the UK Biobank).

Profiling other molecular layers

Metabolomic profiling of EDTA plasma was performed using the Nightingale platform. In the UK Biobank, the Nightingale 2020 assay was measured, whereas the Rotterdam Study and the Leiden Longevity Study used the 2016 assay, which was subsequently re-quantified to align with the Nightingale 2020 assay.[52] Additionally, DNA methylation patterns were measured in the Rotterdam Study, with further details provided elsewhere.[22]

Data splits

In the UK Biobank, we created a 70/20/10 split for all participants, representing the train/test/validation split (n = 28,487/8,138/4,071) for all protein-based biomarkers. We also created a similar split for the subgroup of participants who had both proteomics and metabolomics information available (n = 15,060/4,321/2,053 for train/test/validation splits). We sampled the multi-omic set in such a way that each of the splits for the multi-omics dataset contains only participants of its respective split in the protein-only dataset (i.e., the 15,060 samples in the multi-omics training set are all also within the 28,487 samples of the proteomics-only training set); See Figure S4 for details. We did not stratify on sex, as preliminary experiments (as well as earlier work)[37] show no significant differences between sexes (Table S3).

Ascertainment of covariates and outcomes

Ascertainment of primary outcomes

The Rockwood frailty index (FI), an accumulation measure of age-related deficits,[10,45] was calculated using the previously established methods.[9,53] The FI consisted of 49 deficits in the UK Biobank and 38 in the Rotterdam Study; deficits are detailed in Tables S1 and S2, respectively. The FI was calculated if a person had information on at least 20 deficits before multi-chain imputation as the number of deficits/total number of deficits in that cohort.

All-cause mortality was defined as participants who died from any cause during the total follow-up period, which concluded on the 30th of November 2022 in the UK Biobank, the 5th of July 2024 in the Rotterdam Study, and 8th of April 2024 in the Leiden Longevity Study.

Ascertainment of covariates

Chronological age was calculated as the time between birth and blood sampling. In the UK Biobank, the exact date of birth was not available to researchers; therefore, we used the age at assessment as provided by the UK Biobank. Socioeconomic status was defined as the highest attained education following the UNESCO classification.[54] Current smoking status was defined as a binary

variable (0 = non-smoker, 1 = current smoker) indicating active tobacco use. In all cohorts, weight and length were measured at the research center. Subsequently, body mass index (BMI) was calculated using the established formula weight(kg)/[length(m)].[2] In the Rotterdam Study and UK Biobank, information on cell counts was also available and measured using the percentages of monocytes and lymphocytes.

Ascertainment of secondary outcomes

Having high blood pressure (no = 0, yes = 1) was defined in the UK Biobank by answering "High blood pressure" as one of the answers to the touch screen question: "Has a doctor ever told you that you have had any of the following conditions? (You can select more than one answer)". In the Rotterdam Study and Leiden Longevity Study, high blood pressure was defined as a resting blood pressure exceeding 140/90 mmHg or the use of blood pressure lowering medication. Prevalent cardiovascular disease was defined as a diagnosis prior to blood sampling with a disease with ICD-10 code I20-I25, I48, I50, I60-64, I70, I73, or I48 in the UK Biobank; in the Leiden Longevity Study as I20, I21, or I60–I69, and the Rotterdam Study classification has been described more in depth elsewhere.[55] Incident cardiovascular disease was defined as a first diagnosis with any of these ICD-10 codes after blood sampling and before the end of follow-up: 31st November 2020 in the UK Biobank, 1st January 2020 in the Rotterdam Study, and 1st November 2021 in the Leiden Longevity Study. Participants with prevalent cardiovascular disease were excluded from the incident cardiovascular disease analyses. Maximum hand grip strength was defined as the strength measured using a Jamar hand dynamometer across the two arms in the UK Biobank, the dominant hand in the Leiden Longevity Study, and the non-dominant hand in the Rotterdam Study. Self-reported poor health (no = 0, yes = 1) in the UK Biobank was defined as answering "Poor" or "Fair" to the question "In general how would you rate your overall health?" in the UK Biobank. When a person in the Rotterdam study answered "worse" on the question "How do you rate your health at this moment, compared to your peers" they were seen as having self-rated poor health. In the Leiden Longevity study, answering "Poor" or "Very poor" to "How would you rate your health in general?" was determined as self-rated poor health.

Biomarker models

Linear

ElasticNet regression models were used to construct the linear models. This approach combines Lasso and Ridge regression, allowing for both variable selection and regularization. The loss function that is minimized is:

$$L_{en} = L_{model} + \alpha\left(\beta * ||w||_1 + (1 - \beta) * ||w||_2\right) \quad \text{(Equation 1)}$$

where α and β are regularization hyperparameters used to weigh the L_1 and L_2 loss, and L_{model} depends on whether the model is trained on the frailty index $\left(L^{ft}\|model\right)$ or all-cause mortality $\left(L^{mort}\|model\right)$. In the case of frailty index prediction, constitutes of the mean squared error loss:

$$L^{ft}_{model} = \frac{1}{N_i}\sum_{i=1}^{N}(y_i - \hat{y}_i)^2 \quad \text{(Equation 2)}$$

with N the training set size, y_i the frailty index value for participant i, and $\hat{y}_i$ the estimated frailty index, which is a linear combination of the input: $\hat{y}_i = \sum_{j=1}^{m}\beta x_j$ with m the number of proteins, β_j the scalar pertaining to protein j, and x_j being the protein value of protein j. In the case of mortality prediction, L_{model} is defined by the Cox regression loss:

$$L^{mort}_{model} = \prod_{i \in E_i = 1} \frac{exp(\hat{h})}{\sum_{j \in \{i:T_i \geq t\}} exp(\hat{h})} \quad \text{(Equation 3)}$$

with $\{E_i = 1\}$ the set of participants with an event, and $\{i:T_i \geq t\}$ the set of participants still at risk at time t. $\hat{h}$ is the estimated hazard, defined by the linear combination of proteins as defined by the ElasticNet model.

In the training set, the linear models for the frailty index were built using sklearn's ElasticNetCV v1.3.2. function and the mortality models were developed using sksurv's CoxnetSurvivalAnalysis v0.22.2 with age at censoring as time variable and a binary variable indicating whether the participant was dead at the end of follow-up as event variable.

For both frailty index and mortality prediction, hyperparameter optimization was conducted using 5-fold cross-validation on the training set. A grid search over the β(ranging from 0.1 to 1 in steps of 0.01) was performed. For each β, a list of α values was automatically generated and evaluated to identify the optimal combination of $\beta$and α. For both prediction outcomes, the α-values were logarithmically spaced between a data-driven maximum α_{max} and a minimum α_{min}, where α_{max} defined as the smallest value for which all coefficients are zero, based on the input data[56]:

$$\alpha_{max} = \frac{max(|\langle X, y\rangle|)}{N\beta}$$

We consistently used the function's default settings for α_{min} and α_{max}. For the frailty index models (using ElasticNetCV), α_{min} corresponded to $\alpha_{max} \times 10^{-3}$, whereas for the mortality predictions (using CoxnetSurvivalAnalysis) the default α_{min} corresponded to a

$\alpha_{max} \times 10^{-4}$. Subsequently, the performance of the resulting biomarker models was assessed using mean squared error for frailty index models and concordance index for mortality models. The best model was selected based on lowest error or highest concordance, respectively.

To avoid data leakage, this optimization was nested within the training set (so not touching the test or validation set). The test set was used for the selection of the final models based on performance metrics, while the validation set remained untouched during all modeling decisions. After model selection on the test set, final models were retrained on the combined train and test set (90%) using the previously selected hyperparameters in the nested training on the train set and evaluated on the held-out validation set (10%) to report final performance and biomarker values. Final model parameters can be found in Table S8.

Deep learning

The deep learning models consist of a sequence of alternating linear layers with dropout and reLU nonlinearities. Due to the difference in input dimensionality, slightly different architectures were used for the different protein sets used; See Table S9 for architecture details. All architectures end in a single output neuron, which represents the estimated frailty or log-risk function when trained on the frailty index or mortality, respectively.

The same architectures were used regardless of whether the model was trained on the frailty index or mortality, except for having one extra input neuron when training on frailty index to give age as an input, to match the ElasticNet input. Training on the frailty index uses a regularized mean squared error loss:

$$L_{ft} = \frac{1}{N}\sum_{i \in N}(\hat{y}_i - y_i)^2 + \gamma||W||_2 \quad \text{(Equation 4)}$$

with $\hat{y}_i$ est and y_i being the estimated and true frailty index scores of participant i, and N being the number of training samples. γ is the regularization parameter, with $||W||_2$ the l_2-norm of the model parameters (i.e., weights and biases).

For training an FNN on mortality, following the approach from DeepSurv,[57] the standard Cox partial likelihood loss was transformed into the average negative log partial likelihood:

$$L_{mort} = \frac{-1}{N_{E=1}} \sum_{i \in \backslash\{E_i = 1\backslash\}} \left(\hat{h} - log\left(\sum_{j:\backslash\{i:T_i \geq t\backslash\}} e^{\hat{h}} \right) + \gamma||W||_2 \right) \quad \text{(Equation 5)}$$

with $N_{E=1}$ the number of participants with an observed event, $\hat{h}$ the estimated negative log partial likelihood (i.e., the output of the neural network), $\backslash\{i:T_i \geq t\backslash\}$ being all the participants still at risk at time T_i, and $\gamma||W||_2$ being the same regularization as in Equation 4. All models were implemented using pyTorch.

We used a gridsearch approach to optimize the batch size, learning rate and regularization (γ in Equations 4 and 5) parameters. For the models trained on frailty the gridsearch spanned from 8 to 32, 0.001 to 1.0×10^{-5}, and 0.01 to 1.0×10^{-4} for batch size, learning rate and γ, respectively. For the mortality models, the gridsearch was conducted from 1000 to 20000, 0.001 to 1.0×10^{-6}, and 0.001 to 1.0×10^{-5} for batch size, learning rate and γ, respectively. Final model parameters can be found in Table S10.

While ElasticNet models were optimized using cross-validation, this approach was infeasible for the deep learning models due to the computational complexity. Therefore, the deep learning models were optimized by picking the hyperparameter combination with the highest result on the test (20% hold-out) set (leaving the validation set untouched).

Afterward, our 70/20/10 data split was transformed into a 90/10 data split, retaining the same 10% hold-out validation set and merging the train and test sets. The final models were trained and evaluated on the validation set with the previously determined model parameters. 10 different seeds for parameter initialization were run, and the best model was selected based on either R^2 score or C-index (for the frailty index and all-cause mortality, respectively) in the 90% training set, which was then used for association analysis in the validation set.

Multi-omics

We explored several methods for integrating metabolomics and proteomics for biomarker training: concatenate, principal component analysis (PCA) and then concatenate, combining with MetaboHealth and Angle-Based Joint and Individual Variation Explained (AJIVE). For the concatenate method, we simply concatenated all proteins and metabolites together. For the PCA and then concatenate method, we first took separate PCAs for the protein and metabolomics data, then concatenated the first 10 or 20 principal components. When combining with MetaboHealth, we added the MetaboHealth score to all of the protein features. Finally, AJIVE is a method for identifying shared and separate components from two data modalities.[58,59] Using AJIVE, we identified the shared and individual components of the metabolomics and proteomics data, and used those to train our biomarkers.

Table S5 shows that none of the aforementioned methods increase either the R^2 score or the C-index when trained on the frailty index or mortality, respectively, compared to the baseline using only the cardiometabolic proteins (protein-only). However, this protein-only model was trained on a larger set of participants, due to reduced metabolomics availability. When reducing the training set size for the protein-only model, some deterioration in performance can be seen, which can partially be compensated for by adding metabolites at the input (through concatenation). However, adding more complex multi-omics approaches did not seem to improve performance further.

Forward feature selection

For implementing the forward feature selection procedure, we used the SequentialFeatureSelector function from SKLearn v1.3.2. In short, all features are used to independently create a univariate model to estimate the frailty index (or all-cause mortality). Next, these models are tested using the R^2 score (or C-index). Using a 5-fold nested cross-validation setup on the training set, the protein with the best score metric is selected as the first (most important) protein. In the sub sequent rounds, all linear combinations with the previously selected protein(s) are tested using linear regression, and the best scoring proteins are selected. This process repeats until the scoring metric does not improve more than the tolerance parameter, which was set at 0.001. Note that this procedure only uses the training set, leaving the test and validation set untouched. This resulted in 27 proteins and age selected for the frailty index and 20 for mortality; see Table 2 for the specific proteins.

Calculation of previously developed aging biomarkers

We calculated PAC following the dedicated R-script.[39] The ProteinMortality Score and MetaboMortality Score from Gadd et al.[38] were calculated by multiplying the included proteins and metabolites by their respective weights and calculating a sum score. MetaboHealth was calculated using the MiMIR package.[60] In the Rotterdam Study, we calculated DNAm Hannum, DNAm Horvath using the methylclock R-package[61] and DNAm GrimAge using the Python scripts provided by the researchers who developed these biomarkers.

Association analysis

In order to determine the component of each biomarker independent of age, we created a linear regression predicting chronological age using the biomarker score for each biomarker[62] on the validation set. Then, we determined the residuals of each of these linear regressions, which indicate whether an individual is biologically "older" or "younger" than the population average for their chronological age. After calculating the residuals, to enhance comparison among aging biomarkers, we applied a Z-transformation to these residuals and used this Z-transformed residual in the subsequent analyses with endpoints.

Linear regression analysis using the OLS function from statsmodels v0.14.1 with an added constant was used to assess the associations of the aging biomarkers with continuous outcomes. In case of BMI and smoking, the aging biomarkers were used as the dependent variables in this analysis, as lifestyle factors are hypothesized to influence biological aging, and aging biomarkers are intended to reflect that. Associations of aging biomarkers with binary outcome measures were assessed with logistic regression analyses using the Logit function from statsmodels v0.14.1 with an added constant. Additionally, associations of aging biomarkers with incident outcomes were assessed using Cox-Proportional Hazard models using the CoxPHFitter function from lifelines v0.29.0.

In all analyses, the first model included only sex as a covariate. In the second model, socioeconomic status was additionally added as a covariate. In the third model, next to the covariates from the previous models, BMI and smoking were added as a covariate. In the Rotterdam Study and UK Biobank, we performed analyses in a fourth model and additionally adjusted the analyses for cell counts. Next, we performed the analyses adjusting for the fourth, or in the Leiden Longevity Study third, model with additionally either GDF15 or MetaboHealth to determine whether the associations were independent of these previously established biomarkers. Z-tests were used to assess whether effect sizes between aging biomarkers differed statistically significantly. In a post-hoc sensitivity analysis, we adjusted the all-cause mortality models for cross-sectional frailty index scores to evaluate whether the selected biomarker, ProtFI, contributed prognostic information beyond that provided by frailty alone.

QUANTIFICATION AND STATISTICAL ANALYSIS

Statistical model comparison

To compare the performance of different modeling choices for the aging biomarkers, we applied bootstrap resampling (100 iterations) after completing hyperparameter optimization (on the train (EN) or train and test (FNN) data). This procedure was designed solely to quantify the variability of goodness-of-fit estimates (e.g., R^2 or C-index), not to further optimize the biomarkers. In each iteration, the hyperparameters selected on the original training set were reused. We drew a bootstrap resample from the 90% combined training and test set (sampling participants with replacement) and evaluated model performance on the fixed 10% validation set. Next, to determine whether goodness-of-fit metrics differed significantly between sets, we first assessed the normality of differences using the Shapiro-Wilk test (shapiro function, SciPy v1.12.0). Since all p-values were greater than 0.05, indicating that the differences followed a normal distribution, we proceeded with a paired Z-test assuming unequal variance (ztest function, StatsModels). P-values were false discovery rate adjusted using the Benjamini-Hochberg procedure.[63] A false discovery rate corrected p-value threshold of <0.05 was used to define statistical significance.

Cell Reports Methods, Volume *6*

Supplemental information

ProtFI, an efficient frailty-trained proteomics-based biomarker of aging, robustly predicts age-related decline

Swier Garst, Lieke Kuiper, Erik van den Akker, Niels van den Berg, Mohsen Ghanbari, Simon Mooijaart, Marian Beekman, Marcel Reinders, P. Eline Slagboom, and Joyce van Meurs

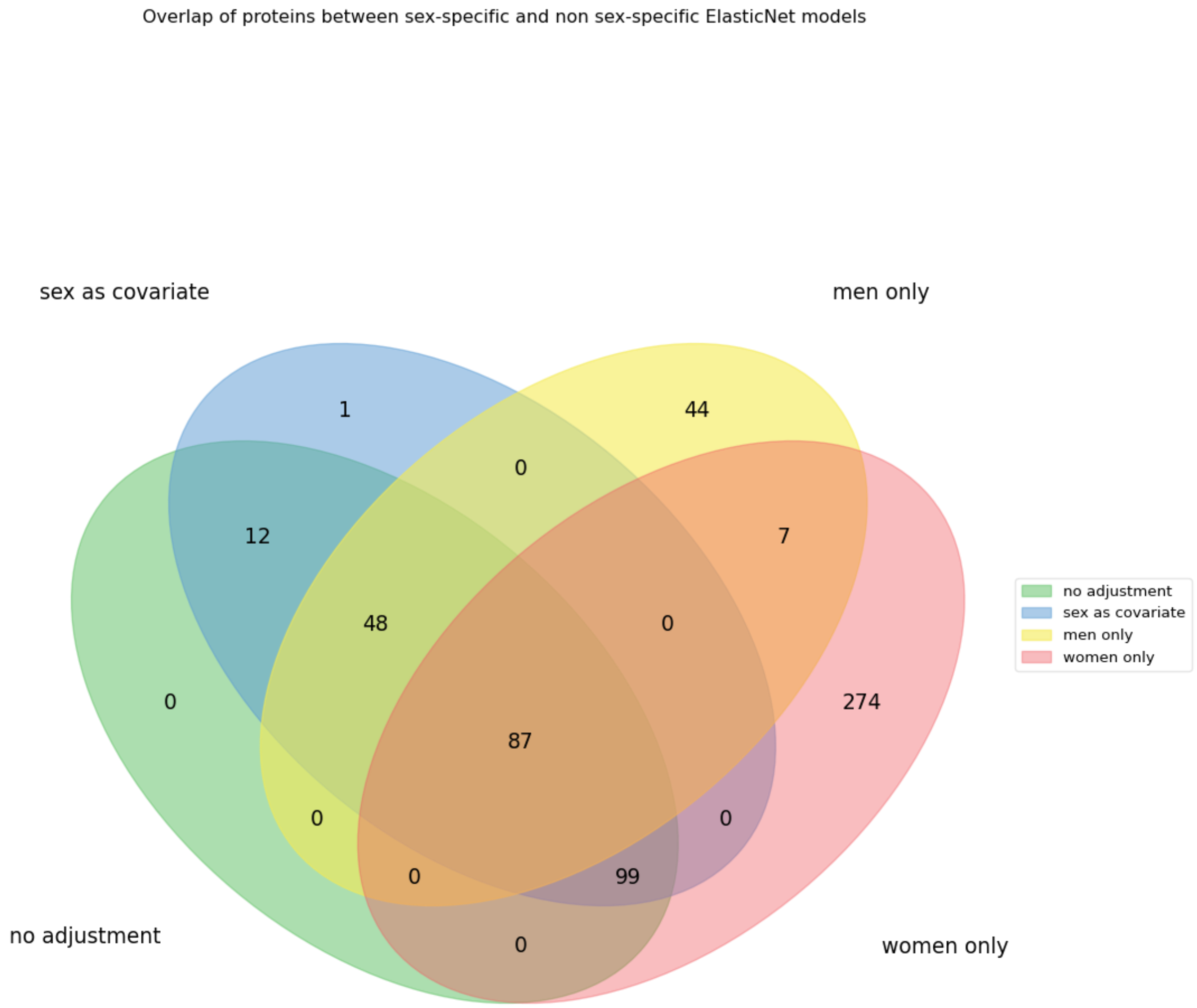

Figure S1. Venn diagram showing the overlap of proteins selected by ElasticNet models for overall mortality across four stratification approaches, related to STAR Methods. ElasticNet models for overall mortality were fitted using four stratification approaches: without sex included, with sex as a covariate, in men only, and in women only. The Venn diagram displays the overlap and unique protein selections obtained from these models.

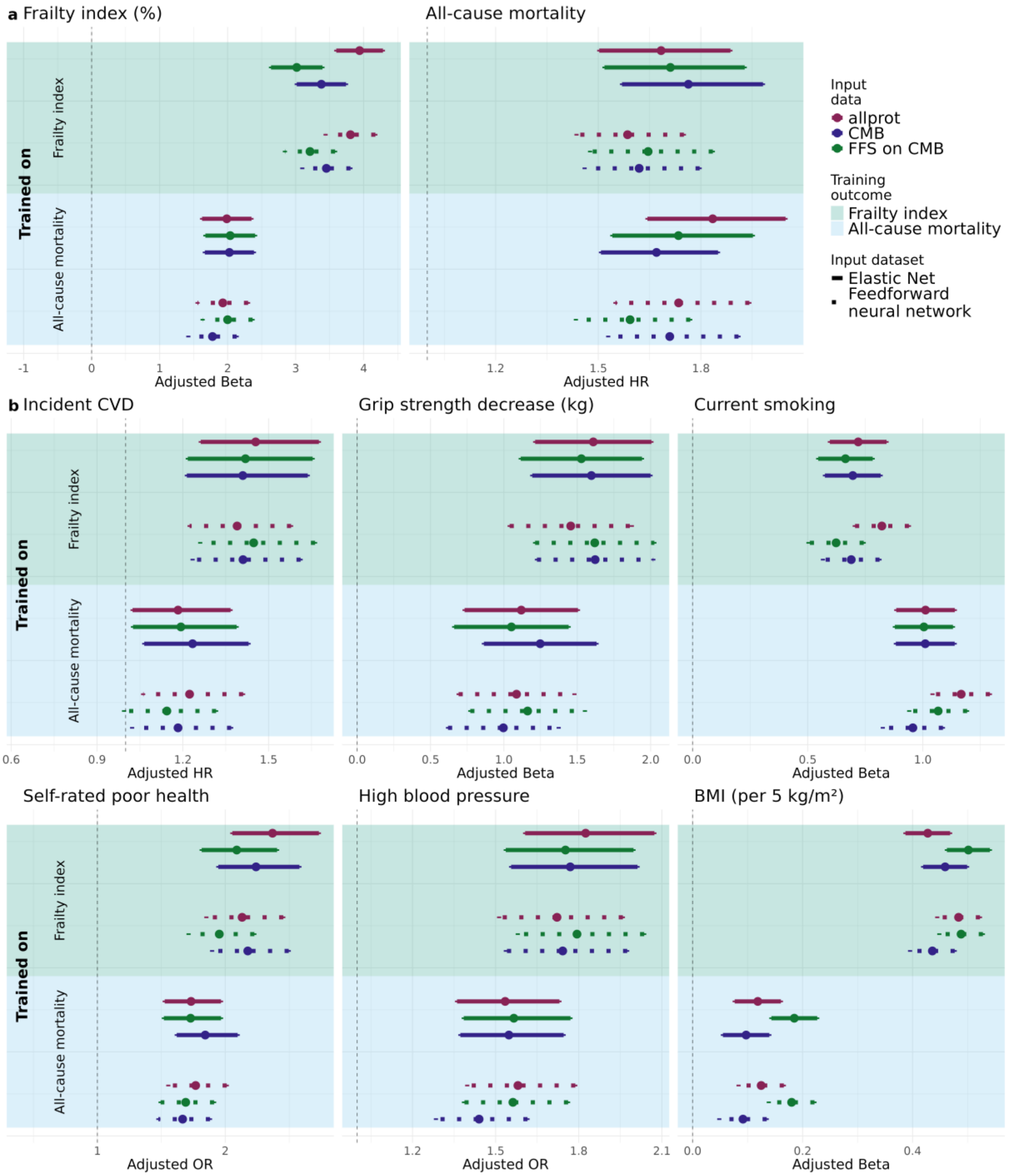

Figure S2. Comparison of biomarkers trained on all proteins, the cardiometabolic panel, and forward feature-selected cardiometabolic proteins for the training endpoints, related to Figures 3 and 4. The figure shows associations of protein-based aging biomarkers trained using the full set of 1,428 proteins from four Olink panels (allprot, red) or the cardiometabolic panel (CMB, blue) or the forward feature selected CMB proteins (FFS) with: A) the training endpoints, namely the frailty index and all-cause mortality, and B) other age-related health indicators. Dots represent the point estimates. Solid and dotted error bars indicate 95% confidence intervals for EN and FNN biomarkers, respectively. The background color indicates the training outcome, with green (upper) for the frailty index and blue (lower) for all-cause mortality. All associations were performed in the independent UKB validation set. See also Table S4.

Allprot indicates the full set of 1428 proteins; CMB the cardiometabolic panel; FFS, forward feature selection; HR, hazard ratio; and OR, odds ratio

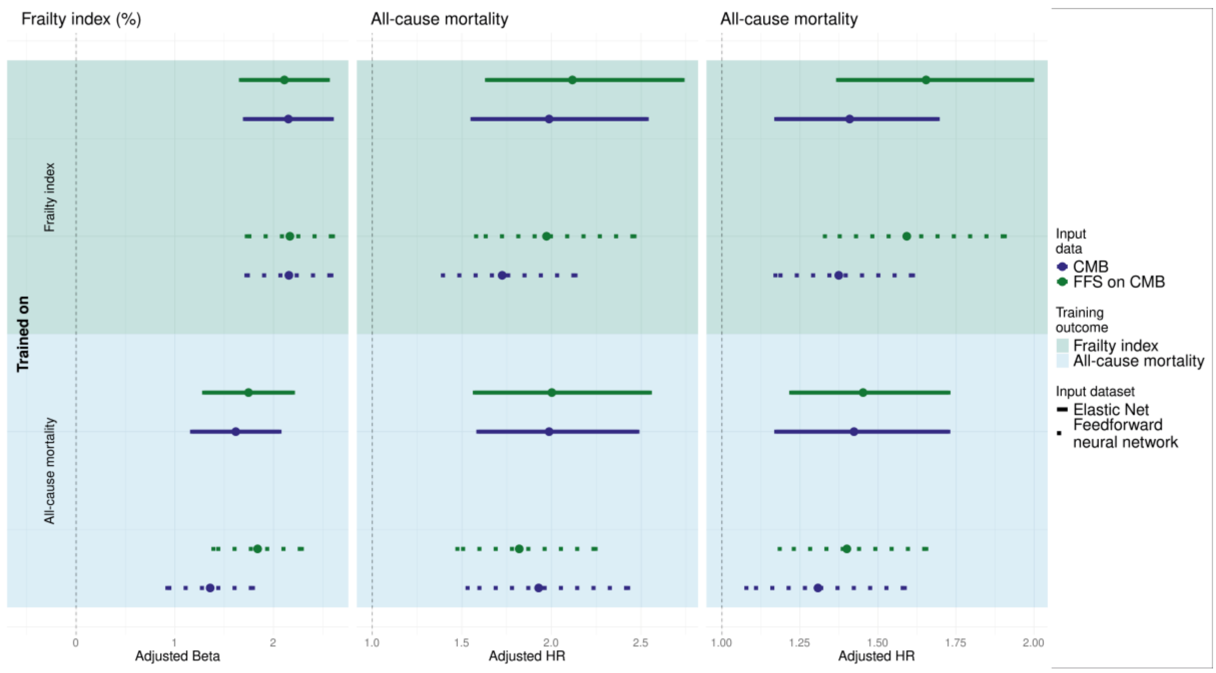

Figure S3. External validation of constructed protein-based aging biomarkers in the Rotterdam Study and the Leiden Longevity Study, related to Figures 4 and 6. The figure shows associations of protein-based aging biomarkers trained using the full set of 1,428 proteins from four Olink panels (allprot, red) or the cardiometabolic panel (CMB, blue) or the forward feature selected CMB proteins (FFS) with: A) the training endpoints, namely the frailty index and all-cause mortality, and B) other age-related health indicators. Dots represent the point estimates. Solid and dotted error bars indicate 95% confidence intervals for EN and FNN biomarkers, respectively. The background color indicates the training outcome, with green (upper) for the frailty index and blue (lower) for all-cause mortality. See also Table S4 and S6.

CMB indicates the cardiometabolic panel; FFS, forward feature selection; and HR, hazard ratio.

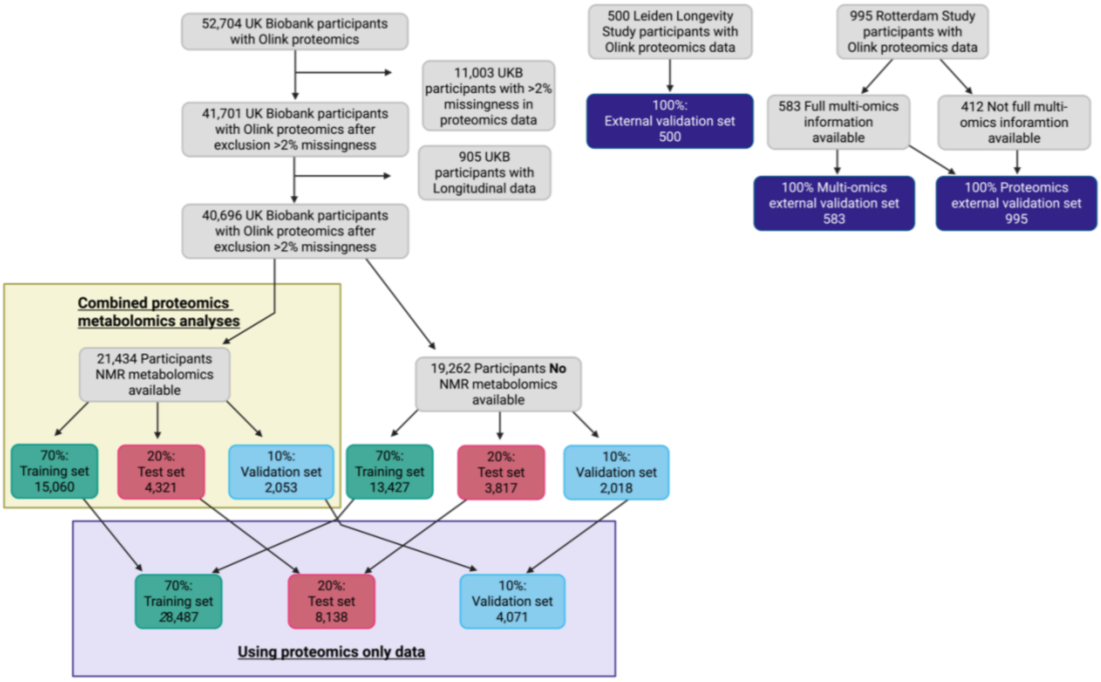

Figure S4. Flowchart of data split creation for the UK Biobank, Leiden Longevity Study, and the Rotterdam Study, related to STAR Methods. This flowchart outlines the creation of training, validation, and external test sets across the UK Biobank, Leiden Longevity Study, and the Rotterdam Study. Participant subsets in the UK Biobank were defined based on availability of proteomics and metabolomics, after which splits were generated for model development and evaluation as detailed in the STAR Methods. The Rotterdam Study dataset consisted of 995 participants with Olink proteomics information with a subset of 583 participants with also information on metabolomics and DNA methylation.

Table S1. The Rockwood frailty index in the UK Biobank, related to STAR Methods.

Type of deficit	Item	Trait	Categories	Coding in Frailty Index
Sensory	1	Glaucoma*	no, yes	Categorized 0/1
	2	Cataracts*	no, yes	Categorized 0/1
	3	Hearing difficulty	no, yes, completely deaf	Categorized 0/1 (combined yes/deaf groups as 1)
Cranial	4	Migraine*	no, yes	Categorized 0/1
	5	Dental problems	ulcers, painful gums, bleeding gums, loose teeth, toothache, dentures	Categorized 0/1 for none vs. any
Mental wellbeing	6	Self-rated health	excellent, good, fair, poor	0 – excellent; 0.25 – good; 0.5 - fair, 1 – poor
	7	Fatigue: frequency of tiredness / lethargy in last two weeks	not at all, several days, more than half, nearly every day	0, 0.25, 0.5, 1, respectively
	8	Sleep: experience of sleeplessness/insomnia	never/rarely, sometimes, usually	Categorized 0, 0.5, 1, respectively
	9	Depressed feelings: frequency in last two weeks	not at all, several days, more than half, nearly every day	0 – not at all, 0.5 – several days, 0.75 -- more than half, 1 – nearly every day
	10	Self-described nervous personality	no, yes	Categorized 0/1
	11	Severe anxiety/ panic attacks*	no, yes	Categorized 0/1
	12	Common to feel loneliness	no, yes	Categorized 0/1
	13	Sense of misery (ever/never)	no, yes	Categorized 0/1
Infirmity	14	Infirmity: long-standing illness or disability	no, yes	Categorized 0/1
	15	Falls in last year	categorical: no falls, one fall, more than one	0, 0.5, 1, respectively
	16	Fractures/broken bones in last five years	no, yes	Categorized 0/1
Cardiometabolic	17	Diabetes*	no, yes	Categorized 0/1
	18	Myocardial infarction*	no, yes	Categorized 0/1
	19	Angina*	no, yes	Categorized 0/1
	20	Stroke*	no, yes	Categorized 0/1
	21	High blood pressure*	no, yes	Categorized 0/1
	22	Hypothyroidism*	no, yes	Categorized 0/1
	23	Deep-vein thrombosis*	no, yes	Categorized 0/1
	24	High cholesterol*	no, yes	Categorized 0/1
Respiratory	25	Breathing: wheeze in last year	no, yes	Categorized 0/1
	26	Pneumonia*	no, yes	Categorized 0/1
	27	Chronic bronchitis/emphysema*	no, yes	Categorized 0/1

	28	Asthma*	no, yes	Categorized 0/1
Musculoskeletal	29	Rheumatoid arthritis*	no, yes	Categorized 0/1
	30	Osteoarthritis*	no, yes	Categorized 0/1
	31	Gout*	no, yes	Categorized 0/1
	32	Osteoporosis*	no, yes	Categorized 0/1
Immunological	33	Hayfever, allergic rhinitis or eczema*	no, yes	Categorized 0/1
	34	Psoriasis*	no, yes	Categorized 0/1
Cancer	35	Any cancer diagnosis*	no, yes	Categorized 0/1
	36	Multiple cancers diagnosed (number reported)	Range from 0 to 6	0 - no cancer or single cancer, 1 - multiple cancers
Pain	37	Chest pain	no, yes	Categorized 0/1
	38	Head and/or neck pain	no, yes (combining responses to pain in head and neck/shoulders)	Categorized 0/1
	39	Back pain	no, yes	Categorized 0/1
	40	Stomach/abdominal pain	no, yes	Categorized 0/1
	41	Hip pain	no, yes	Categorized 0/1
	42	Knee pain	no, yes	Categorized 0/1
	43	Whole-body pain	no, yes	Categorized 0/1
	44	Facial pain	no, yes	Categorized 0/1
	45	Sciatica*	no, yes	Categorized 0/1
Gastrointestinal	46	Gastric reflux*	no, yes	Categorized 0/1
	47	Hiatus hernia*	no, yes	Categorized 0/1
	48	Gall stones*	no, yes	Categorized 0/1
	49	Diverticulitis*	no, yes	Categorized 0/1

* indicates self-reported
The Rockwood frailty index in the UK Biobank, as developed by Williams et al.[1], following the procedure of Searle et al.[2]. The Rockwood frailty index in the UK Biobank consist of 49 items, coded according to the table above. The frailty score is calculated by summing all 49 codes and dividing by the total number of items.

Table S2. The Rockwood frailty index in the Rotterdam Study, related to STAR Methods.

Trait	Item	Trait(s) – additional information	Cutoff value	Based on
Dressing and grooming	1	Able to get clothes from closets or drawers; able to dress; able to shampoo your hair; able to comb your hear or do your make up	Without any difficulty = 0<br>With some difficulty = 0.33<br>With much difficulty = 0.66<br>Unable to do = 1	Stanford Health Assessment Questionnaire
Arising	2	Able to stand up from a straight chair without using your arms for support; able to get in and out of bed	Without any difficulty = 0<br>With some difficulty = 0.33<br>With much difficulty = 0.66<br>Unable to do = 1	Stanford Health Assessment Questionnaire
Eating	3	Able to cut meat and lift a full cup or glass to your mouth; able to open a new carton of milk	Without any difficulty = 0<br>With some difficulty = 0.33<br>With much difficulty = 0.66<br>Unable to do = 1	Stanford Health Assessment Questionnaire
Walking	4	Able to walk outdoors on flat ground; able to climb up five steps	Without any difficulty = 0<br>With some difficulty = 0.33<br>With much difficulty = 0.66<br>Unable to do = 1	Stanford Health Assessment Questionnaire
Hygiene	5	Able to wash and dry your entire body; able to take a shower/bath	Without any difficulty = 0<br>With some difficulty = 0.33<br>With much difficulty = 0.66<br>Unable to do = 1	Stanford Health Assessment Questionnaire
Reach	6	Able to reach and get down a 1kg object from just above your head; able to bend down to pick up clothing from the floor	Without any difficulty = 0<br>With some difficulty = 0.33<br>With much difficulty = 0.66<br>Unable to do = 1	Stanford Health Assessment Questionnaire
Grip	7	Able to open a car door? Able to open jars which have been previously opened	Without any difficulty = 0<br>With some difficulty = 0.33<br>With much difficulty = 0.66<br>Unable to do = 1	Stanford Health Assessment Questionnaire
Riding a bike	8	Able to ride a bike	Without any difficulty = 0<br>With some difficulty = 0.33<br>With much difficulty = 0.66<br>Unable to do = 1	Lawton Instrumental Activities of Daily Living scale
Telephone	9	Able to use the telephone	Without any difficulty = 0<br>With some difficulty or using a customized phone = 0.33<br>With much difficulty = 0.66<br>Unable to do = 1	Lawton Instrumental Activities of Daily Living scale
Meal	10	Able to prepare meals	Without any difficulty = 0<br>With some difficulty = 0.33<br>With much difficulty = 0.66<br>Unable to do = 1	Lawton Instrumental Activities of Daily Living scale
Medication	11	Able to take in medication	Without any difficulty = 0<br>With some difficulty = 0.33<br>With much difficulty = 0.66<br>Unable to do = 1	Lawton Instrumental Activities of Daily Living scale
Laundry	12	Able to do the laundry	Without any difficulty = 0<br>With some difficulty = 0.33<br>With much difficulty = 0.66<br>Unable to do = 1	Lawton Instrumental Activities of Daily Living scale
Financial	13	Able to do finances	Without any difficulty = 0<br>With some difficulty = 0.33<br>With much difficulty = 0.66<br>Unable to do = 1	Lawton Instrumental Activities of Daily Living scale

Depressed affect	14	I felt that I could not shake off the blues even with help from family or friends; I felt depressed; I thought my life had been a failure; I felt lonely; I had crying spells; I felt sad	Rarely or none of the time = 0<br>Some or a little of the time = 0.33<br>Occasionally or a moderate amount of time = 0.66<br>Most or all of the time = 1	The CES-D scale: a self-report depression scale
Positive affect	15	I felt that I was just as good as other people; I felt hopeful about the future; I was happy; I enjoyed life	Rarely or none of the time = 1<br>Some or a little of the time = 0.66<br>Occasionally or a moderate amount of time = 0.33<br>Most or all of the time = 0	The CES-D scale: a self-report depression scale
Somatic and retarded activity	16	I did not feel like eating my appetite was poor; I had trouble keeping my mind on what I was doing felt that everything I did was an effort; I felt fearful; my sleep was restless; I talked less than usual; I could not get “going”	Rarely or none of the time = 0<br>Some or a little of the time = 0.33<br>Occasionally or a moderate amount of time = 0.66<br>Most or all of the time = 1	The CES-D scale: a self-report depression scale
Interpersonal	17	I was bothered by things that usually don’t bother me; people were unfriendly; I felt that people dislike me	Rarely or none of the time = 0<br>Some or a little of the time = 0.33<br>Occasionally or a moderate amount of time = 0.66<br>Most or all of the time = 1	The CES-D scale: a self-report depression scale
Falling	18	How often did you fell the past 12 months?	No falling = 0<br>Less than once a month = 0.5<br>More than once a month = 1	
Joint complaints	19	Did you have joint pain or other complaints from the knees, hips, back or hand?	No = 0<br>Yes = 1	
Forgetfulness	20	Do you sometimes forget what you was about to do?	No = 0<br>Yes = 1	
Aphasia	21	Do you have difficulties with finding the right words?	No = 0<br>Yes = 1	
Liver enzymes	22	ALAS, ALAT, Gamma-glutamyl transpeptidase	All values within the range = 0<br>One or more abnormal values = 1	Serum blood measurement; cutoff values derived from the Laboratory guide Erasmus MC
Creatinine	23		Male 65-115 umol/L = 0<br>Other values = 1<br>Female 55-90 umol/L = 0<br>Other values = 1	Serum blood measurement; cutoff values derived from the Laboratory guide Erasmus MC
Hyperlipidemia	24	High cholesterol or medication against high cholesterol	Statin use and/or cholesterol >6.5 mmol/L<br>No statin use and cholesterol 2.9-6.5 mmol/L	Serum blood measurement; cutoff values derived from the Laboratory guide Erasmus MC
HDL	25		HDL ≥ 1.55 = 0<br>HDL < 1.55 = 1	Serum blood measurement; cutoff values derived from the Laboratory guide Erasmus MC

Systolic blood pressure	26	Measure three times, average is taken	Systolic blood pressure 90-140 = 0<br>Systolic blood pressure 140-160 = 0.5<br>Systolic blood pressure < 90 = 0.5<br>Systolic blood pressure > 160 = 1	Serum blood measurement; cutoff values derived from the Laboratory guide Erasmus MC
MMSE	27	Mini Mental State Examination	Unimpaired >25 = 0<br>Impaired ≤ 25 =1	
LDST	28	Letter-Digit Substitution Test: the number of correct digits	Above mean or less than 1SD below mean = 0<br>One SD below mean = 0.5<br>Two SD below mean = 1	
STROOP	29	Stroop test	Above mean or less than 1SD above mean = 0<br>One SD above mean = 0.5<br>Two SD above mean = 1	
WFT	30	Word Fluency test	Above mean or less than 1SD below mean = 0<br>One SD below mean = 0.5<br>Two SD below mean = 1	
Cancer	31		No = 0<br>Yes = 1	
Lung condition (COPD/Asthma)	32		No = 0<br>Yes = 1	
Cardiovascular diseases	33		No = 0<br>Yes = 1	Prevalent CHD
Stroke	34		No = 0<br>Yes = 1	Prevalent stroke
Diabetes Mellitus	35		No = 0<br>High glucose= 0.5<br>Yes = 1	Prevalent DM and/or glucose levels
BMI	36	Body mass index<br>BMI < 18.5 = underweight<br>BMI ≤25 and ≥18.5 = normal weight<br>BMI <25 and ≤30 = overweight<br>BMI <30 = obese	Normal weight= 0<br>Overweight = 0.5<br>Obese or underweight = 1	
Age-related macular degeneration	37	Fundus photography after pharmacologic mydriasis. The eyes of each participant were graded and classified separately, and the eye with the more severe grade was used to classify the person.	0 = 5-year risk of developing advanced AMD in at least one eye is 0.5%<br>0.25 = 5-year risk is 3%<br>0.50 = 5-year risk is 12%<br>0.75 = 5-year risk is 25%<br>1= 5-year risk is 50%	
Hospital admission	38	Last 12 months	No = 0<br>Yes = 1	

The Rockwood frailty index in the Rotterdam Study, as developed by Schoufour et al.[3], following the procedure of Searle et al.[2]. The Rockwood frailty index in the Rotterdam Study consist of 38 items, coded according to the table above. The frailty score is calculated by summing all 38 codes and dividing by the total number of items.

Table S3. Concordance indexes on the four-protein panel (allprot) dataset using various adjustments for sex, related to STAR Methods.

	Linear	Deep
No adjustment	0.73	0.73
Sex as (input) covariate	0.73	0.73
Men only	0.72	0.70
Women only	0.71	0.72

Table S5. Goodness-of-fit metrics on various multi-omics integration methods, related to STAR Methods.

Dataset / method	ElasticNet		Feedforward Neural Networks	
	Frailty index (R^2)	All-cause mortality (C)	Frailty index (R^2)	All-cause mortality (C)
Using only cardiometabolic proteins	0.24	0.71	0.25	0.71
Cardiometabolic proteins in population subset with metabolomics	0.23	0.69	0.20	0.68
Cardiometabolic proteins and NMR-metabolites concatenated	0.24	0.69	0.23	0.69
Using AJIVE	0.17	0.61	0.14	0.62
PCA then concatenate, using 10 principal components	0.17	0.61	0.16	0.64
PCA then concatenate, using 20 principal components	0.19	0.67	0.17	0.66
Cardiometabolic proteins and MetaboHealth, concatenated	0.23	0.69	0.23	0.68

AJIVE indicates Angle-based Joint and Individual Variation Explained; C, concordance index; NMR, nuclear magnetic resonance; PCA, principal component analysis.

Table S8. Hyperparameters for the ElasticNet models, related to STAR Methods.

	Frailty index		All-cause mortality	
	α	β	α	β
Allprot	2.5×10^{-4}	0.99	1.6×10^{-3}	0.99
CMB	1.5×10^{-4}	0.99	8.4×10^{-3}	0.1
FFS	2.2×10^{-5}	0.99	7.6×10^{-4}	0.69

Allprot indicates, all protein dataset; CMB, cardiometabolic panel; and FFS, proteins resulting after forward features selection

Table S9. Neural network architectures, related to STAR Methods.

	Layer 1	Layer 2	Layer 3	Layer 4
Allprot	Linear: 1428*x 750<br>Dropout: p = 0.2<br>ReLU	Linear: 750 x 400<br>Dropout: p = 0.1<br>ReLU	Linear: 400 x 100<br>Dropout: p = 0.1<br>ReLU	Linear: 100 x 1
CMB	Linear: 344*x 200<br>Dropout: p = 0.2<br>ReLU	Linear: 200 x 100<br>Dropout: 0.1<br>ReLU	Linear: 100 x 10<br>ReLU	Linear: 10 x 1
FFS	Linear: 20-28** x 15<br>ReLU	Linear: 15 x 10<br>ReLU	Linear: 10 x 5<br>ReLU	Linear: 5 x 1

*input dimension is 1 higher when trained on frailty, to give age as an input
**input dimension is 20 for mortality-trained, and 28 for frailty-trained models.

Table S10. Model hyperparameters for the Feedforward Neural Networks, related to STAR Methods.

	Frailty index			All-cause mortality		
	Batch	Learning	γ	Batch	Learning	γ
Allprot	16	5.0×10^{-6}	$5.0 \times 10^{-}$[illegible]	1000	5.0×10^{-6}	$1.0 \times 10^{-}$[illegible]
CMB	22	1.0×10^{-5}	$1.0 \times 10^{-}$[illegible]	2000	5.0×10^{-5}	$5.0 \times 10^{-}$[illegible]
FFS	17	1.0×10^{-4}	$1.0 \times 10^{-}$[illegible]	4060	6.3×10^{-4}	$1.0 \times 10^{-}$[illegible]

Allprot indicates, all protein dataset; CMB, cardiometabolic panel; and FFS, proteins resulting after forward features selection

Supplementary references

1. Williams, D.M., Jylhävä, J., Pedersen, N.L., and Hägg, S. (2019). A Frailty Index for UK Biobank Participants. Journals of Gerontology: Series A *74*, 582–587. https://doi.org/10.1093/gerona/gly094.
2. Searle, S.D., Mitnitski, A., Gahbauer, E.A., Gill, T.M., and Rockwood, K. (2008). A standard procedure for creating a frailty index. BMC Geriatr. *8*, 24. https://doi.org/10.1186/1471-2318-8-24.
3. Schoufour, J.D., Erler, N.S., Kiefte-de Jong, J.C., Kiefte-de Jong, J.C., Voortman, T., Ziere, G., Lindemans, J., Klaver, C.C., Tiemeier, H., Stricker, B., et al. (2017). Design of a frailty index among community living middle-aged and older people: The Rotterdam study. Maturitas *97*, 14–20. https://doi.org/10.1016/j.maturitas.2016.12.002.
